# Targeting intracellular LMP2 with costimulatory signal–armed antibody-like TCR T cells

**DOI:** 10.1172/jci.insight.178572

**Published:** 2025-05-22

**Authors:** Jiali Cheng, Xuelian Hu, Zhenyu Dai, Yuhao Zeng, Jin Jin, Wei Mu, Qiaoe Wei, Xiangyin Jia, Jianwei Liu, Meng Xie, Qian Luo, Guang Hu, Gaoxiang Wang, Xiaojian Zhu, Jianfeng Zhou, Min Xiao, Jue Wang, Taochao Tan, Liang Huang

**Affiliations:** 1 Department of Hematology, Tongji Hospital, Tongji Medical College of Huazhong University of Science and Technology, Wuhan, China.; 2 Immunotherapy Research Center for Hematologic Diseases of Hubei Province, Wuhan, China.; 3 Department of Hematology, The First Affiliated Hospital of Zhejiang University, Zhejiang University, Hangzhou, China.; 4 Department of Internal Medicine, Cleveland Clinic, Akron General, Cleveland, Ohio, USA.; 5 Division of General Internal Medicine, Department of Medicine, University of Pittsburgh Medical Center, Pittsburgh, Pennsylvania, USA.; 6 IASO Biotherapeutics, Nanjing, China.; 7 State Key Laboratory of Experimental Hematology, National Clinical Research Center for Blood Diseases, Haihe Laboratory of Cell Ecosystem, Institute of Hematology & Blood Diseases Hospital, Chinese Academy of Medical Sciences & Peking Union Medical College, Tianjin, China.; 8 Tianjin Institutes of Health Science, Tianjin, China.

**Keywords:** Infectious disease, Therapeutics, Cancer immunotherapy, Immunotherapy

## Abstract

Expanding the repertoire of CAR therapies to include intracellular antigens holds promise for treating a broad spectrum of malignancies. TCR-like T cells, capable of recognizing intracellular antigen–derived peptides in complex with HLA molecules (pHLA), represent a promising strategy in the field of engineered cellular therapy. This study introduced antibody-like TCR (abTCR) T cells that specifically targeted HLA-A*02:01–restricted LMP2_426_ peptides, a typical Epstein-Barr virus (EBV) latency II protein, for the treatment of EBV-associated lymphoproliferative diseases (EBV-LPDs). Compared with classic CAR T cells targeting the same epitope, abTCR T cells demonstrated superior efficiency, including increased CD107A expression, enhanced cytotoxicity, and elevated IFN-γ secretion, even when engaging with target cells that naturally present antigens. Moreover, a costimulatory signal–armed abTCR (Co-abTCR), which integrated a costimulatory structure with the abTCR, further enhanced the proliferation and in vivo tumoricidal efficacy of transfected T cells. Collectively, our study developed a potentially novel TCR-like T cell therapy that targets HLA-A*02/LMP2_426_ for the treatment of EBV-LPDs, providing a potential therapeutic solution for targeting of intracellular antigens in cancer immunotherapy.

## Abstract 2

We developed a novel Co-abTCR T-cell, potent to treat EBV-associated diseases and instructive in cellular therapies targeting intracellular antigens.

## Introduction

The lack of suitable therapeutic targets remains a major challenge in cancer treatment, as most tumor-specific antigens (TSAs) in tumors are intracellular and not directly targetable. Human leukocyte antigen–presented (HLA-presented) TSA–derived peptides (pHLA) offer a promising alternative. However, despite decades of research, technical hurdles persist in the development of immunotherapies effectively targeting these pHLAs.

There are primarily 2 main approaches to target pHLA: utilizing natural T cell receptors (TCRs) (1) or engineering TCR-like antibodies (2). The infusion of in vitro–expanded, tumor-specific natural TCR T cells has shown therapeutic promise in conditions such as Epstein-Barr virus–associated (EBV-associated) posttransplant lymphoproliferative disorders (PTLD). However, this approach is limited by time-intensive manufacturing processes (3). Deciphering the sequence of a pHLA-specific TCR allows for rapid and stable production of pHLA-specific T cells through gene-engineering techniques. However, this approach carries the risk of mispairing with endogenous TCRs. Moreover, the efficacy of TCR T cells may be hampered by the insufficient binding affinity of natural TCRs to pHLA (generally 1 × 10^–4^ to 1 × 10^–6^ M), due to the negative selection process during T cell development in the thymus (4).

In contrast, pHLA-specific antibodies, often referred to as TCR-like antibodies, usually have high binding affinities. The great success of CAR T cells designed to target membrane proteins has sparked the development of TCR-like CAR T cells that target pHLA. For instance, Rafiq et al. engineered HLA-A*02:01/WT1–specific TCR-like CAR T cells to treat WT1-overexpressing carcinomas (5). However, CAR T cells typically require antigen densities 10–100 times higher than those needed for TCR T cells to achieve full activation (6, 7). Notably, natural density of specific pHLA complexes on cell membranes is exceedingly low, 1.3–9 molecules per cell (8, 9). But effective initiation of CAR T cell responses is suggested to require over 200 molecules per cell (10). This substantial difference presents a formidable challenge when targeting pHLA expressed on normal cells with TCR-like CAR T cells.

EBV contributes to various hematological disorders, including PTLD (11), hemophagocytic lymphohistiocytosis (12), and lymphomas (13, 14). Nearly 40% of non-Hodgkin lymphomas are associated with EBV infection, with certain subtypes, such as T/NK cell lymphoma, showing a 100% causative association (15). The latency II protein LMP2 is widely expressed in EBV-associated diseases, rendering it an ideal therapeutic target. Moreover, HLA-A*02:01, referred to as A*02, is a commonly shared HLA type globally (16), and peptides CLGGLLTMV derived from LMP2 (LMP2_426_) and presented by A*02, have been targeted using TCR-T or single-chain variable fragment–based (scFv-based) antibody approaches, providing concrete evidence that HLA-A*02:01/LMP2_426_ is a promising therapeutic target (2, 17). However, no clinically viable strategies have emerged to date.

Recently, Xu et al. reported an antibody-like TCR (abTCR) structure that integrates a high-affinity Fab domain for antigen recognition with intracellular domains of γ (UniProtKB [https://www.uniprot.org/]: locus TRGC1_HUMAN, accession P0CF51) and δ (UniProtKB: locus TRDC_HUMAN, accession B7Z8K6) chains of the TCR for activation signaling (18). This design provides an improved theoretical framework for engineering T cells to target low-density pHLA complexes. Building on this concept, our research aimed to develop optimally engineered T cells that target HLA-A*02:01/LMP2_426_, referred to as A*02/LMP2, for treatment of EBV-associated diseases. Using the abTCR structure as a foundation, we conducted a comprehensive investigation that included (a) screening antibodies specific to A*02/LMP2 from a fully human phage library; (b) evaluating efficacy and specificity of abTCR, CAR and TCR mimic structures, and (c) optimizing abTCR by incorporating a costimulatory domain. To our knowledge, this study marks the pioneering application of an optimized abTCR structure in T cell immunotherapy targeting an intracellular protein.

## Results

### Screening and functionality verification of A*02/LMP2-specific abTCR T cells.

**Figure 1:**
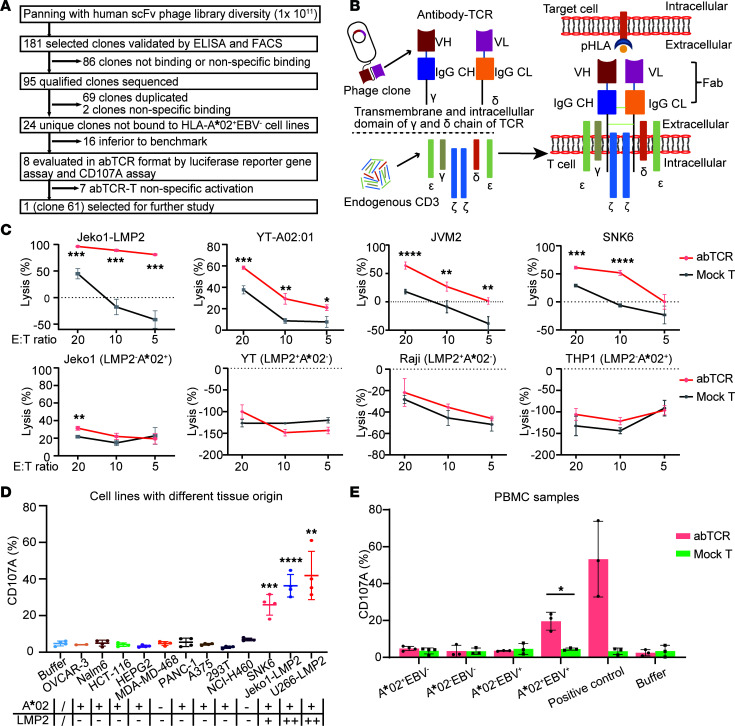
Screening and verification of A*02/LMP2-specific abTCR T cells. (**A**) Schematic of the screening and selection process. (**B**) Schematic diagram of abTCR and its functional format abTCR/CD3 complex. VH/VL, the variable domain of the heavy chain/light chain obtained from candidate phage clones; IgG CH and IgG CL, the constant domain 1 of the heavy chain and light chain of IgG; γ/δ, the transmembrane and intracellular domains of the γ/δ chain of TCR. Engineered abTCR consists of the 2 chains, *VH-IgG CH-*γ and *VL-IgG CL-*δ, which assemble and form complex with endogenous CD3 on T cell membrane to exert functions. (**C**) Cytolysis of 61abTCR T cells (red line) and mock T cells (gray line) against target-positive tumor cells, including Jeko1-LMP2 (LMP2_426_ overexpressed by PresentER minigene system), YT-A*02:01 (HLA-A*02:01 exogenously expressed), JVM2, and SNK6, as well as target-negative tumor cells, including Jeko1, YT, Raji, and THP1, at the specified E/T ratio for approximately 24 hours. Results from 3 or 4 replicates, with mean ± SD shown. Student’s *t* test was used for the statistical analysis. (**D**) CD107A expression on CD8^+^abTCR^+^ cells after overnight coincubation with cell lines of different tissue origins (summarized in Supplemental Table 1). The HLA-A*02 phenotype and LMP2 expression was indicated at the bottom of the figure. +, positive; ++, overexpressed; –, negative. The mean ± SD from 2–4 different donors are shown. Student’s *t* test was used for the statistical analysis. (**E**) CD107A expression on CD8^+^abTCR^+^ cells (red column) and CD8^+^CD3^+^ mock T cells (green column) after overnight incubation with healthy donor PBMC, including A*02^+^ and A*02^–^, and EBV-infected (copy number of EBV per 2 × 10^5^ cells greater than 1 × 10^5^) donor-derived PBMC, including A*02^+^ and A*02^–^. Jeko1-LMP2 or T2, pulsed with 0.5 μg/mL LMP2_426_, and buffer were set as positive and negative controls. Jeko1-LMP2–pulsed cells were used as the positive control, and buffer-treated cells served as the negative control. Sample size: 3–4. Each sample was tested once. Paired *t* test was used. *P < 0.05; **P < 0.01; ***P < 0.001; ****P < 0.0001.

The screening steps of A*02/LMP2-specific phage clone were depicted in Figure 1A. A total of 181 enriched phage clones from the humanized scFv phage library were tested by ELISA and FACS to identify clones positive for A*02/LMP2 and negative for A*02/WT1_129_ or TNF-α, and 95 qualified clones were then sequenced. Finally, 24 unique clones were obtained. Compared with the previously reported A*02/LMP2-specific E38 clone (2), 8 clones exhibited comparable binding performance (Supplemental Figure 1, A and B; supplemental material available online with this article; https://doi.org/10.1172/jci.insight.178572DS1). These 8 lead candidates were further examined in abTCR format (Figure 1B and Supplemental Figure 1C). By conducting a LUC gene reporter assay in Jurkat–nuclear factor of activated T cell–firefly LUC–abTCR (Jurkat-NFAT-ffLuc-abTCR) cells, and CD107A assay in abTCR T cells (Supplemental Figure 1D and Supplemental Figure 2), clone 61 emerged as the top candidate due to its favorable activity and specificity.

We first characterized the binding parameters of 61rFc, a recombinant fusion protein consisting of Fab region based on clone 61 and Fc region of rabbit IgG. The parameters were as follows: (a) The dominant binding epitope of nonameric peptide (LMP2_426_) positioned at 5(L), 7(T), and 8(M), identified by sequential alanine scanning mutagenesis assay (Supplemental Figure 3A). (b) The binding affinity was 11.9 nM, similar to E38 of 10.5 nM (Supplemental Figure 3B). (c) EC_50_ was 40.19 nM when incubated with LMP2-peptide–pulsed T2 cells (Supplemental Figure 3C).

We subsequently assessed the functionality of 61abTCR (abTCR structure with VH and VL domain derived from clone 61) T cells. Compared with mock T cells, 61abTCR T cells displayed robust antitumor effects against Jeko1-LMP2 cells (80% versus –121%, effector-to-target [E/T] ratio of 1, *P* < 0.0001), which artificially present A*02/LMP2 through LMP2_426_ peptides encoding PresentER minigene system (Supplemental Figure 4, A and C), and a moderate effect against JVM2 cells (24.7% versus –39.0%, E/T ratio of 10, *P* < 0.0001), SNK6 cells (51.7% versus –6.3%, E/T ratio of 10, *P* < 0.0001), and YT-A*02:01 cells (29.3% versus 8.6%, E/T ratio of 10, *P* < 0.0001), which presented A*02/LMP2 through endogenous natural processing (Supplemental Figure 4B). However, there was no observed effect on LMP2^–^ or A*02^–^ cells (Figure 1C). Furthermore, we evaluated the degranulation process in 61abTCR T cells upon tumor cell stimulation using the CD107A assay. The average CD107A expression on CD8^+^abTCR^+^ T cells was found to be 25.9%, 36.3%, and 41.9% after stimulation with SNK6, Jeko1-LMP2, and U266-LMP2 (LMP2_426_ overexpressed by the PresentER minigene system), respectively, compared with 4.8% in the effector-only group (Figure 1D). This finding suggests successful activation of abTCR T cells. Importantly, no cross-reactivity was observed, as the CD107A expression levels on TCR T cells remained comparatively low when cocultured with antigen-negative cell lines from various tissues or healthy donor PBMC (regardless of the A*02 phenotype) (Figure 1, D and E, and Supplemental Table 1). Moreover, abTCR T cells were specifically activated by EBV^+^/A*02^+^ PBMCs but not by EBV^+^/A*02^–^ PBMCs (Figure 1E). These data support the functionality of abTCR T cells toward tumor cells in an antigen-dependent manner.

### AbTCR T cells outperformed mTCR T cells and 3rdCAR T cells in vitro.

**Figure 2:**
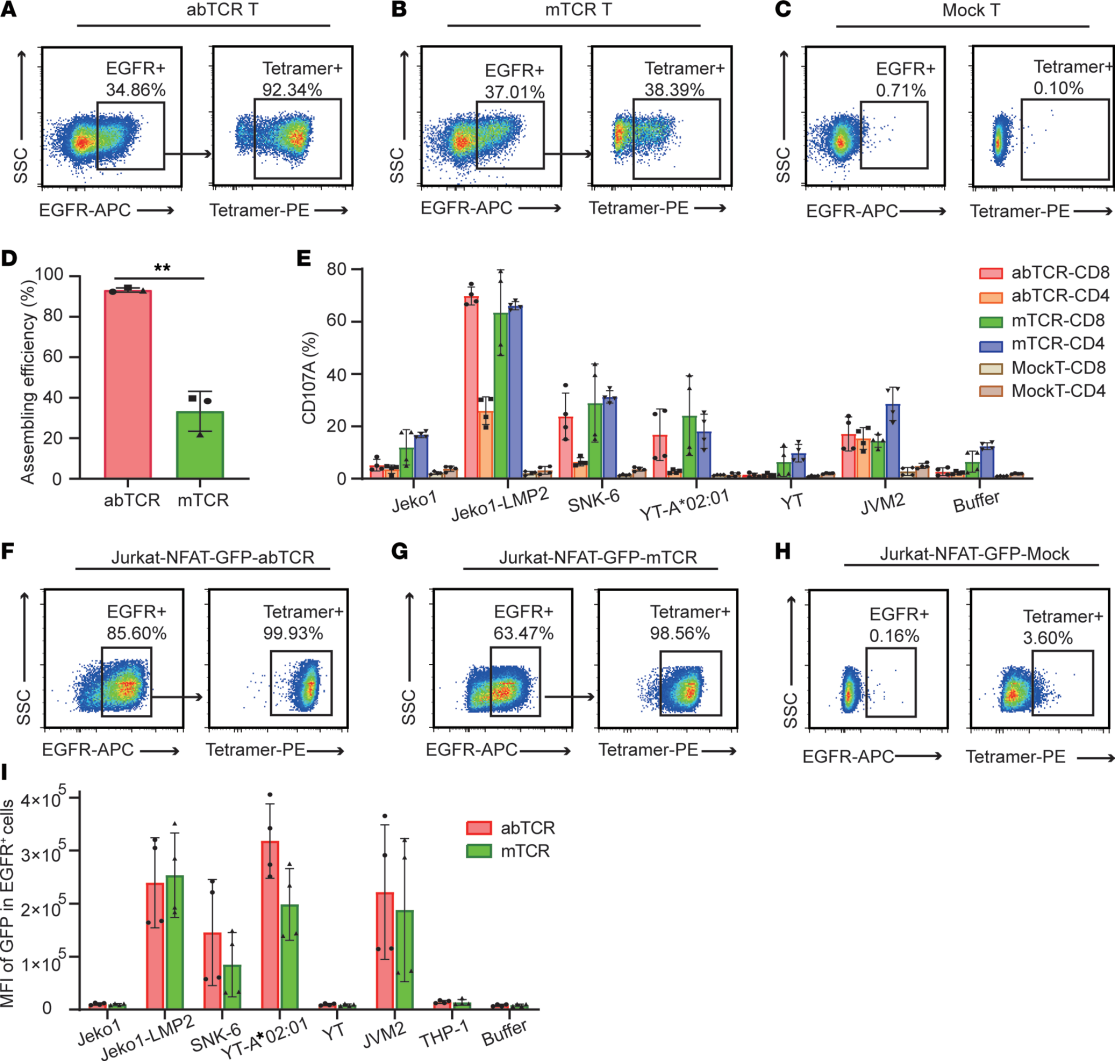
The comparison between abTCR and mTCR. (**A**–**C**) Representative plots showing the binding rate to the HLA-A*02:01/LMP2 tetramer in abTCR^+^ T cells (**A**), mTCR^+^ T cells (**B**), and mock T cells (**C**) obtained by flow cytometry. (**D**) The comparison of the assembly efficiency in abTCR T cells and mTCR T cells, with data from 3 biological repeats. Paired *t* test was used. (**E**) The expression of CD107A in CD4^+^EGFR^+^ and CD8^+^EGFR^+^ cells in abTCR T cells, mTCR T cells, or mock T cells in response to the indicated cell lines stimulation. Results were from 4 replicates. (**F**–**H**) Flow cytometry results showing the binding rate to the A*02/LMP2 tetramer in Jurkat-NFAT-GFP reporter cells transfected with abTCR (**F**), mTCR (**G**), and mock (**H**). (**I**) The MFI of GFP in EGFR^+^ cells in Jurkat-NFAT-GFP-abTCR cells (red column) and Jurkat-NFAT-GFP-mTCR cells (green column) stimulated by the indicated cell lines. The experiments repeated 4 times. Mean ± SD were plotted for this figure. **P < 0.01.

The functions of abTCR T cells were compared with the mimic TCR (mTCR) T cells and third-generation CAR (3rdCAR) T cells, all derived from clone 61 (Supplemental Figure 5). The mTCR was modeled after the natural αβTCR structure, with only the variable domain of α and β chains replaced by the variable domain of light and heavy chains of 61 antibody (Supplemental Figure 5, A, B, and E). The mean assembly efficiency of mTCR in T cells was only 33.33% (SD ± 9.91%), significantly lower than the 93.23% (SD ± 1.04%) observed for abTCR (Figure 2, A–D). This suggests that exogenously expressed mTCR is prone to endogenous mismatches, potentially causing nonspecific activation. CD107A assay further confirmed this hypothesis. In the presence of negative target cells (Jeko1, YT) and even without target cells (buffer group), CD4^+^ mTCR T cells and CD8^+^ mTCR T cells showed higher expression of the degranulation marker CD107A, specifically at 16.73%, 9.77%, and 12.46% for CD4^+^ cells and 11.97%, 6.4%, and 6.50% for CD8^+^ cells. These values markedly exceeded those of the mock T control group, whereas abTCR T cells did not exhibit such nonspecific activation. Upon stimulation with target-positive cells (Jeko1-LMP2, SNK6, YT-A*02:01, JVM2), CD8^+^ abTCR T cells demonstrated a pronounced upregulation of CD107A expression, comparable with CD8^+^ mTCR T cells, indicating similar sensitivity between abTCR and mTCR (Figure 2E). Furthermore, we transfected mTCR or abTCR into Jurkat-NFAT-GFP reporter cells, resulting in high assembly efficiencies of 99.93% for mTCR and 98.56% for abTCR, respectively (Figure 2, F–H). This high assembly efficiency in this model minimized the likelihood of mismatch-induced nonspecific activation. Upon antigen stimulation, both abTCR and mTCR demonstrated the ability to mediate the activation of the NFAT signaling pathway, with abTCR eliciting a more pronounced, though not statistically significant, activation (Figure 2I). These results suggest that abTCR may trigger T cell activation more effectively. Overall, compared with mTCR, abTCR structure exhibited superior specificity and sensitivity.

**Figure 3:**
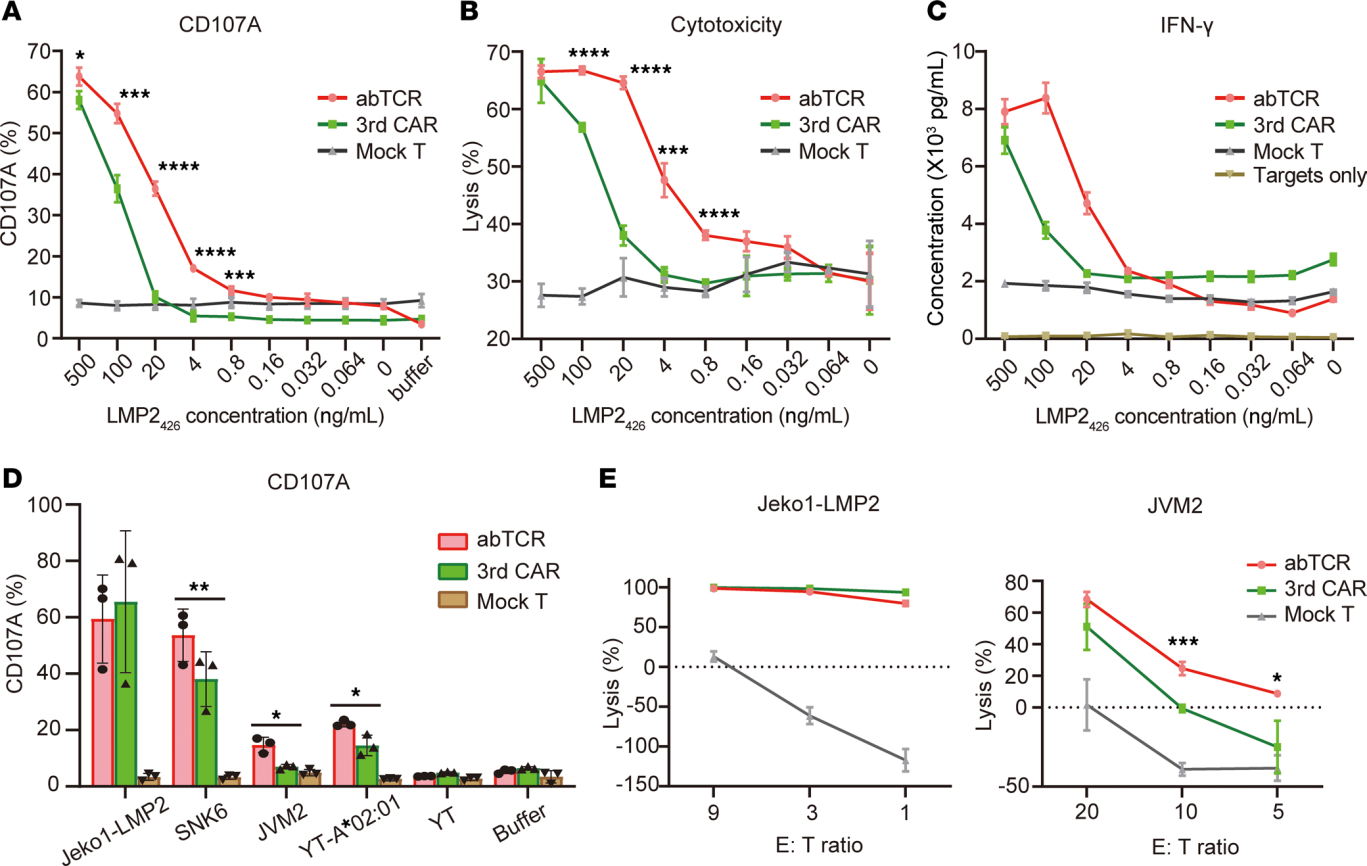
Comparing the functionality of abTCR T cells and 3rdCAR T cells. (**A**) CD107A expression on CD8^+^abTCR^+^ cells (red line), CD8^+^3rdCAR^+^ cells (green line), and CD8^+^CD3^+^ mock T cells (gray line) after overnight incubation with T2 cells pulsed with different concentration of LMP2_426_ peptides. *n* = 3. (**B**) Percentage of necrotic (PI^+^) target cells (T2-pulsed with different concentration LMP2_426_ peptides) after 24-hour coculture with the effector cells (abTCR/3rdCAR T cells) or controls (mock T cell/buffer). *n* = 3. For **A** and **B**, the mean ± SD are shown. One-way ANOVA with Bonferroni’s correction for multiple comparison was performed. A significant difference between abTCR group and 3rd CAR group was marked only when the value in the abTCR T group is significantly higher than that in mock T group. (**C**) IFN-γ concentration in the supernatant after 24-hour coculture of effector cells/controls with T2 cells pulsed with a different concentration of LMP2_426_ peptides. *n* = 2. (**D**) CD107A expression on CD8^+^abTCR^+^ cells, CD8^+^3rdCAR^+^ cells, and CD8^+^CD3^+^ mock T cells upon stimulation from different cell lines, specified on the *x* axis. *n* = 3. One-way ANOVA for matched data design with Bonferroni’s correction for comparison between abTCR group and 3rd CAR was used. (**E**) Cytolysis of abTCR/3rdCAR/mock T cells toward Jeko1-LMP2 and JVM2 at the specified E/T ratios. Results are from 3 replicates. One-way ANOVA with Bonferroni’s correction for multiple comparison was performed. *P < 0.05, **P < 0.01, ***P < 0.001, ****P < 0.0001.

We than compared abTCR T cells with 3rdCAR T cells (Supplemental Figure 5, C–E). In a T2-loading model, the CD107A expression level, cytolytic effect, and secretion of IFN-γ in 3rdCAR T cells and abTCR T cells all decreased as the concentration of loaded LMP2_426_ peptides decreased (Figure 3, A–C). This indicates that the activity of abTCR or 3rdCAR T cells is dependent on antigen density. Interestingly, under low LMP2_426_ peptide concentration conditions, abTCR T cells displayed superior functionality compared with 3rdCAR T cells, indicating a higher sensitivity of abTCR. These findings were validated in cell lines. When responding to naturally presented antigens, such as SNK6, JVM2, and YT-A*02:01 cells, abTCR T cells were better activated than 3rdCAR (Figure 3D). However, in response to artificially overexpressed antigens, such as in Jeko1-LMP2, both abTCR T cells and 3rdCAR T cells displayed pronounced and comparable activation (Figure 3D). Similar results were observed in the cytolysis assay against Jeko1-LMP2 and JVM2 (Figure 3E). These results collectively suggest that abTCR outperformed 3rdCAR in response to naturally presented pHLA antigens.

### AbTCR T cells exhibited limited in vivo proliferative potency.

Next, we tested the in vivo antitumor activity of abTCR T cells in NCG mice incubated with JVM2 cells (Supplemental Figure 6A). No significant survival benefits were observed (Supplemental Figure 6B). The weight of the mice remained stable across all groups, suggesting well-tolerated treatments (Supplemental Figure 6C). After abTCR T cell infusion, the proportion of abTCR^+^ T cells within CD3^+^ T cells initially increased slightly but dropped dramatically after day 7, indicating limited abTCR^+^ T cell expansion (Supplemental Figure 6D). Moreover, we examined the proliferation of abTCR T cells in vitro through a repeated antigen stimulation assay. After 3 rounds of stimulation, abTCR T cells showed minimal response to JVM2 but demonstrated profound proliferation against Jeko1-LMP2 (Supplemental Figure 6E). These differences may be attributed to the varied A*02/LMP2 density on target cells.

### Costimulatory signaling enhanced the potency of abTCR T cells in vitro.

**Figure 4:**
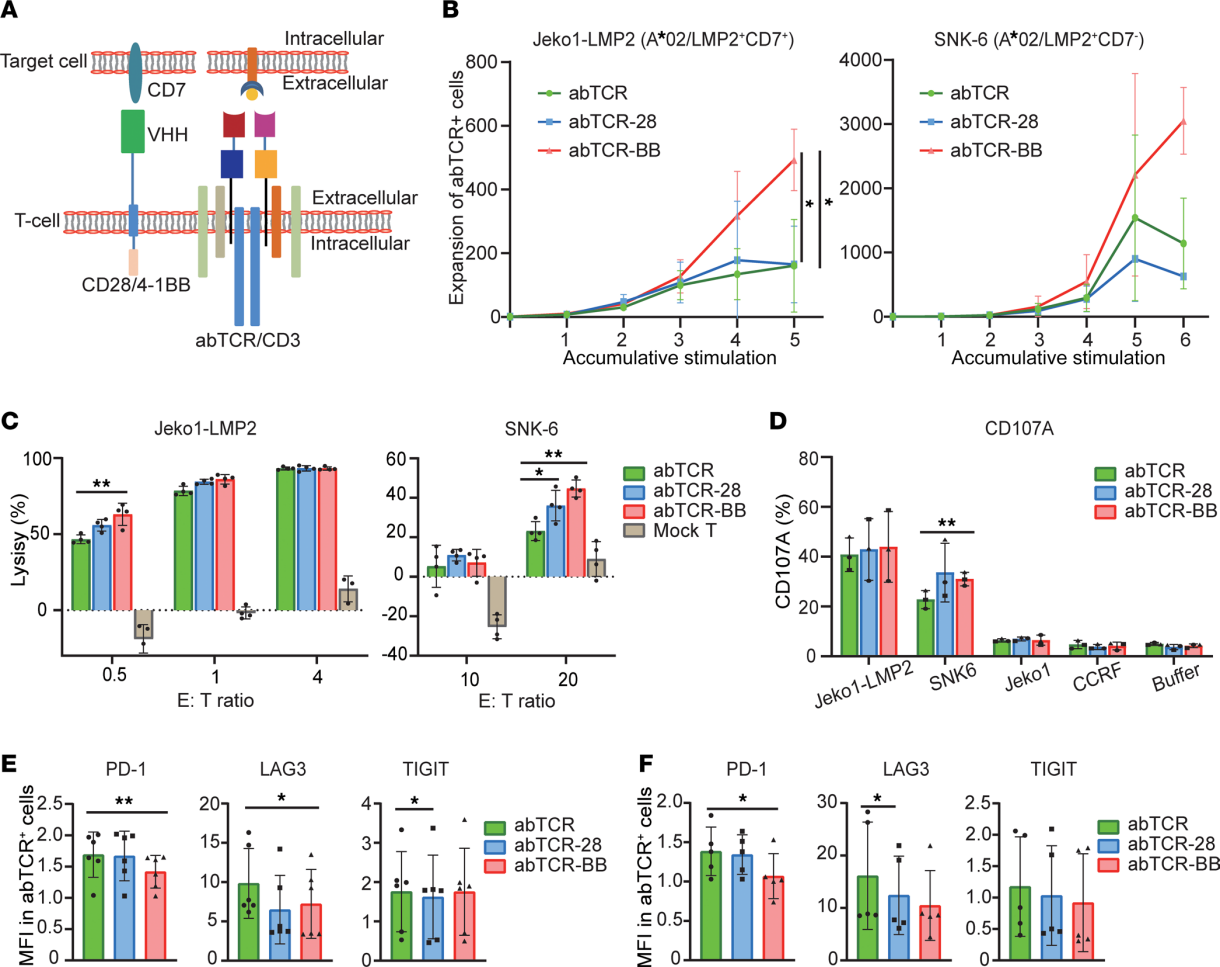
Optimization of abTCR T cells. (**A**) Schematic diagram of optimized abTCR T cells. A chimeric receptor, composed of CD7-specific VHH, transmembrane domain and costimulatory domain, and CD28 or 4-1BB, is introduced into abTCR T cells, which can provide costimulatory signals, CD28, or 4-1BB upon CD7 stimulation. (**B**) The accumulative expansion folds of abTCR^+^, abTCR-28^+^, and abTCR-BB^+^ T cells after repeated stimulation of Jeko1-LMP2 cells (A*02/LMP2^+^CD7^+^) or SNK6 (A*02/LMP2^+^CD7^–^). The mean ± SD from 5 independent replicates were presented. One-way ANOVA for matched data design followed by Bonferroni’s correction for multiple comparison was used. The ANOVA test for SNK6 stimulation was significant (*P* = 0.0258). (**C**) Representative cytolysis to Jeko1-LMP2 and SNK6 cells of abTCR T cells, abTCR-28 T cells, abTCR-BB T cells, and mock T cells from 1 donor, 3–4 repeats. One-way ANOVA with Bonferroni’s correction for comparison between abTCR-28/abTCR-BB T cells and abTCR T cells was performed. (**D**) The CD107A expression on CD8^+^ abTCR^+^, abTCR-28^+^, and abTCR-BB^+^ cells after incubation with the *x* axis–specified cell lines. The means ± SD from 3 different donors were presented. One-way ANOVA for matched data design with Bonferroni’s correction for comparison between abTCR-28/abTCR-BB group and abTCR group was performed. (**E** and **F**) The MFI of the exhaustion markers PD1, LAG3, and TIGIT on abTCR^+^, abTCR-28^+^, and abTCR-BB^+^ cells after repeated stimulation with Jeko1-LMP2 (**E**) or SNK6 (**F**). Mean ± SD from 5 or 6 independent experiments were plotted. One-way ANOVA for matched data design with Dunnett’s correction for comparison between abTCR-28/abTCR-BB group and abTCR group was performed. *P < 0.05; **P < 0.01.

To enhance the potency of abTCR T cells, we introduced costimulatory signaling of CD28 or 4-1BB into the abTCR T cells (19, 20). We incorporated the costimulatory signaling by including an anti-CD7 nanobody (21), linked to either CD28 (abTCR-28) or 4-1BB (abTCR-BB) domain (Figure 4A and Supplemental Figure 7A). The expression rates of abTCR, abTCR-28, and abTCR-BB on T cells and of CD7 on different target cell lines are shown in Supplemental Figure 7, B and C. As a result, the proliferation of abTCR-BB T cells was profoundly enhanced, with an average of a 10.12-fold increase in cell numbers when stimulated with Jeko1-LMP2 (CD7^+^) and a 2.03-fold increase when stimulated with SNK6 (CD7^–^) (Figure 4B). Furthermore, abTCR-BB T cells displayed greater tumoricidal efficacy than abTCR T cells, as evidenced by a higher lysis rate against Jeko1-LMP2 (63.1% versus 46.5%, E/T ratio of 1:2, *P* = 0.0069) and SNK6 (44.7% versus 23.1%, E/T ratio of 20, *P* = 0.0013) (Figure 4C). Similar trends were observed in abTCR-28 T cell groups, albeit less pronounced. Furthermore, abTCR-BB T cells stimulated with SNK6 showed a statistically significant increase in CD107A expression (31.0% versus 22.7%, *P* = 0.008) (Figure 4D). The antitumor activity and CD107A expression of abTCR-BB or abTCR-28 T cells were comparable with those of mock T cells when cocultured with pHLA^–^CD7^+^ Jeko1 and CCRF cells, suggestive of target specificity (Supplemental Figure 7D and Figure 4D). Intriguingly, following repeated stimulation with Jeko1-LMP2 and SNK6, the expression of exhaustion markers PD1, LAG3, and TIGIT remained at relatively low levels on abTCR-BB T cells in comparison with abTCR T cells (Figure 4, E and F). Together, incorporation of costimulatory signaling (4-1BB superior to CD28) remarkably improved the proliferation, cytolysis, and degranulation of abTCR T cells, without promoting exhaustion.

### AbTCR T cells exhibited tumoricidal effects against artificially overexpressed antigens in vivo.

**Figure 5:**
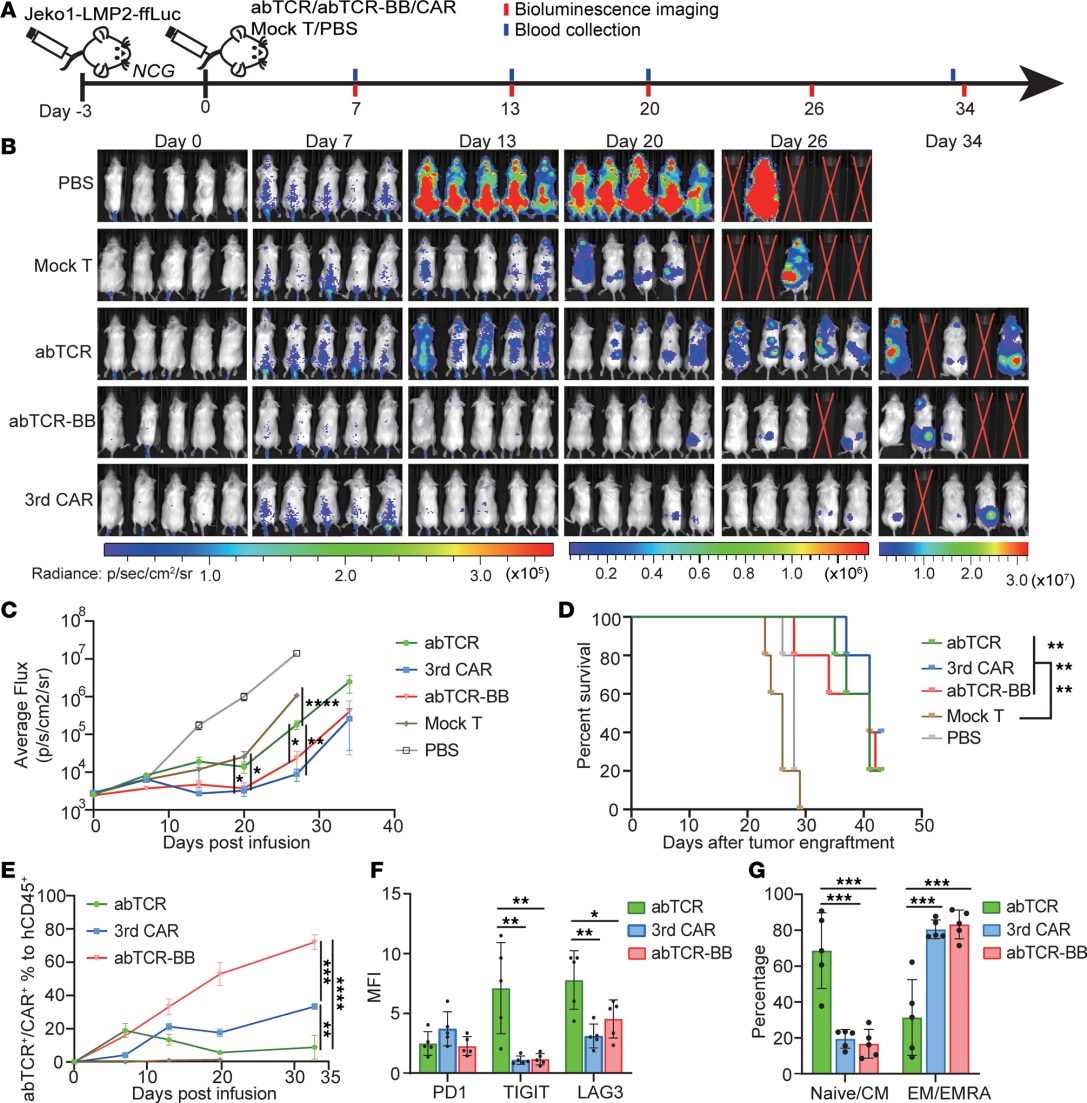
The functionality of abTCR, 3rdCAR, and Co-abTCR T cells in Jeko1-LMP2 xenografted mice model. (**A**) The experimental design. NCG mice were i.v. injected with 1 × 10^6^ Jeko1-LMP2-ffLuc cells at day –3, and treated by 4 × 10^6^ abTCR T cells, 3rdCAR T cells, Co-abTCR T cells, mock T cells, or PBS at day 0. Bioluminescence imaging and blood collection were performed as the indicated time. (**B**) The bioluminescence imaging analysis of mice in different groups on days 0, 7, 13, 20, 26, and 34. The crossed red lines indicate that the mouse was dead. (**C**) The tumor burden, presented by average radiance, of mice in each group over time. The significance * at day 20 referred to 3rdCAR versus mock T, and abTCR-BB versus mock T, by multiple comparison with Bonferroni’s correction after 1-way ANOVA. Only the comparison at day 20 between 3rdCAR or abTCR-BB versus mock T was specifically highlighted. (**D**) The survival of mice in different groups over time. Log-rank test was used. (**E**) The percentage of abTCR^+^ or CAR^+^ cells to hCD45^+^ cells in the peripheral blood of mice over time. One-way ANOVA with Bonferroni’s correction for multiple comparison was used. (**F** and **G**) The MFI of exhaustion markers, PD1, TIGIT, and LAG3 (**F**), and the percentage of naive (CD45RO^–^CCR7^+^)/central memory (CD45RO^+^CCR7^+^) or effector memory (CD45RO^+^CCR7^–^)/terminally differentiated effector cells (CD45RO^–^CCR7^–^) (**G**) in abTCR^+^, 3rdCAR^+^, or Co-abTCR^+^ cells at 20 days after infusion. Data were all presented as mean ± SEM. One-way ANOVA with Bonferroni’s correction for multiple comparison test was used. *P < 0.05; **P < 0.01; ***P < 0.001; ****P < 0.0001.

The activity of abTCR, 3rdCAR, and abTCR-BB (hereafter referred to as Co-abTCR) T cells was tested in NCG mice i.v. xenografted with Jeko1-LMP2-ffLuc cells, characterized by artificially overexpressed antigens (Figure 5A). The tumor burden in mice treated with 3rdCAR T cells or Co-abTCR T cells was significantly lower than that in the mock-T and PBS control groups at day 20 after infusion, and the significance was not identified for the abTCR T cell group until 26 days after infusion (Figure 5, B and C). Co-abTCR T cells and 3rdCAR T cells demonstrated comparable antitumor efficacy, while the efficacy was superior to abTCR T cells (Figure 5, B and C). Although mock T cells initially displayed modest tumor growth inhibition, their effect gradually diminished at 20 days. By 27 days after infusion, the control groups exhibited a 100% fatality rate. In contrast, all mice in the treatment groups survived except for 1 mouse in the Co-abTCR group that died despite having a low tumor burden. Autopsies revealed no noticeable abnormalities. All abTCR T cells, Co-abTCR T cells, and 3rdCAR T cells significantly extended the survival of Jeko1-LMP2–bearing mice compared with mock T cells (median survival days, 41/41/41 versus 26, *P* = 0.0021/0.0007/0.0021) (Figure 5D). The proportion of abTCR^+^ in hCD45^+^ cells consistently increased in the Co-abTCR and 3rdCAR group (Figure 5E), supporting their better proliferative potential than abTCR T cells, whose percentage declined after day 7. Moreover, Co-abTCR T cells exhibited more robust expansion compared with 3rdCAR T cells (Figure 5E). The exhaustion markers TIGIT and LAG3 were lower on Co-abTCR^+^ T cells and 3rdCAR^+^ T cells compared with abTCR^+^ T cells at 20 days after infusion (Figure 5F and Supplemental Figure 8). Meanwhile, the abTCR group had a higher percentage of naive (CD45RO^–^CCR7^+^) and central memory (CD45RO^+^CCR7^+^) phenotypic cells compared with Co-abTCR and 3rdCAR groups, whereas effector memory (CD45RO^+^CCR7^–^) and terminally differentiated effector memory (CD45RO^–^CCR7^–^) T cells were more abundant in the Co-abTCR and 3rdCAR group (Figure 5G and Supplemental Figure 8). This suggested full activation and differentiation of Co-abTCR^+^ and 3rdCAR^+^ cells in response to Jeko1-LMP2 stimulation in vivo.

In conclusion, all abTCR, 3rdCAR, and Co-abTCR T cells exhibited antitumor efficacy against Jeko1-LMP2 cells in vivo and significantly prolonged the survival of mice. Co-abTCR T cells and 3rdCAR T cells showed superior tumor control and expansion compared with abTCR T cells in this model.

### Co-abTCR exhibited superior expansion and antitumor efficacy against endogenously presented antigens in vivo.

**Figure 6:**
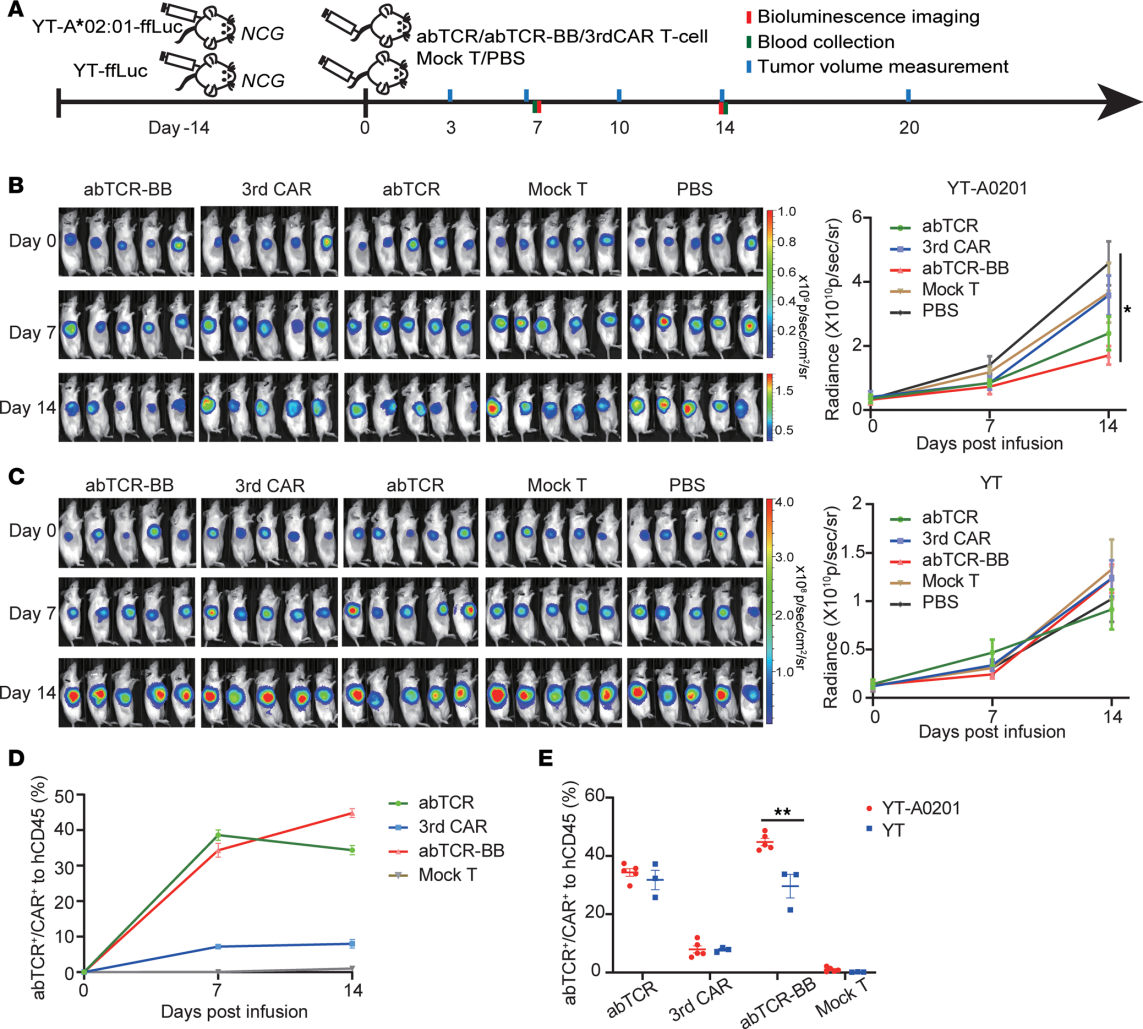
The functionality of abTCR, 3rdCAR, and abTCR-BB T cells in YT-A*02:01 xenografted mice model. (**A**) The experimental design. NCG mice were s.c. implanted with 5 × 10^6^ YT-A*02:01-ffLuc cells or YT-ffLuc cells at day –14 and treated by 4 × 10^6^ abTCR T cells, 3rdCAR, Co-abTCR T cells, mock T cells, or PBS at day 0, when the tumor volume exceeded 100 mm^3^. Bioluminescence imaging, tumor volume measurement, and blood collection were performed as the indicated time. (**B** and **C**) Tumor burden monitored by bioluminescence imaging over time in YT-A*02:01–bearing (**B**) or YT-bearing (**C**) mice. One-way ANOVA with Bonferroni’s correction for multiple comparison test was used. (**D**) The percentage of abTCR^+^, 3rdCAR^+^, or Co-abTCR^+^ in hCD45^+^ cells over time. (**E**) The comparison of the percentage of abTCR^+^, 3rdCAR^+^, or Co-abTCR^+^ in hCD45^+^ cells between YT-A*02:01 model and YT model at day 14 after treatment. In the YT-ffLuc model, only 3 mice were randomly selected to collect peripheral blood. Student’s *t* test was used. The mean ± SEM was plotted. *P < 0.05; **P < 0.01.

We further examined the therapeutic effect of abTCR, 3rdCAR, and Co-abTCR T cells in NCG mice s.c. xenografted with YT-A*02:01-ffLuc, which endogenously present antigens. Parental YT-ffLuc cells served as a negative control (Figure 6A). In the YT-A*02:01-ffLuc model, tumor burdens as monitored by bioluminescence imaging were significantly lower in mice treated with Co-abTCR T cells than in PBS group 14 days after treatment (Figure 6B). While the abTCR group exhibited a reduction in tumor burden compared with control groups, statistical significance was not observed. For the 3rdCAR group, no antitumor efficacy was observed when compared with mock T group at day 14 (Figure 6B). Additionally, Co-abTCR T cells significantly controlled tumor volumes at 14 days (versus mock T group) and 20 days (versus mock T and PBS groups) after treatment (Supplemental Figure 9). As expected, none of the abTCR, 3rdCAR, or Co-abTCR T cells had a therapeutic effect on our YT- ffLuc NCG mice models (Figure 6C and Supplemental Figure 9), verifying their specificity. All mice in abTCR T cell/3rdCAR T cell/control groups were euthanized, whereas only 1 in the Co-abTCR group was euthanized due to a large tumor volume (>2,000 mm^3^) at day 20. We monitored the percentage of abTCR^+^, CAR^+^, and Co-abTCR^+^ T cells in hCD45^+^ cells in the mice’s blood after infusion, and we observed a notable and persistent increase of the Co-abTCR percentage but a transient increase of the abTCR and CAR percentage (Figure 6D). Comparing the results between the YT-A*02:01 model and the YT model at 14 days after treatment, we found that the percentage of abTCR^+^ cells was similar in the 2 groups, indicating that no specific expansion of abTCR T cells occurred in the YT-A*02:01-ffLuc mice group (Figure 6E). The same result was identified in the 3rdCAR group (Figure 6E). On the other hand, the percentage of Co-abTCR^+^ cells was significantly higher in the YT-A*02:01-ffLuc mice group compared with the YT-ffLuc group (44.8% versus 29.6%, *P* = 0.004), indicative of the specific expansion of Co-abTCR T cells in response to YT-A*02:01-ffLuc (Figure 6E). Collectively, this demonstrated that the incorporation of 4-1BB signals enhanced the antitumor efficacy and expansion in vivo in response to endogenously presented A*02/LMP2 targets; however, its capability to fully eradicate tumors remains suboptimal.

## Discussion

T cells engineered to target intracellular antigens with cytolytic potential represent a promising therapeutic avenue in cancer immunotherapy. In this study, we developed potentially novel Co-abTCR T cells targeting A*02/LMP2 for the treatment of EBV-associated diseases. Unlike the previously reported A*02/LMP2-targeted E38 bispecific antibody, we employed a cellular immunotherapy approach, capable of robust proliferation and long-term persistence. A key focus was the development and optimization of a chimeric receptor construct suitable for TCR-like T cell therapy. The costimulatory CAR-armed antibody-like TCR construct was ultimately identified as the most effective, having the following advantages: (a) utilization of fully human abTCR structures, which have higher sensitivity than 3rdCAR and better specificity than mTCR, well-suited for detecting low-density antigens, and (b) incorporation of a costimulatory signaling pathway to substantially enhance functional capabilities.

The specificity of engineered TCR-like T cells requires rigorous evaluation. Targeted peptides, typically spanning 8–14 aa, constitute only 2%–3% of the aa within the entire pHLA complex (22, 23). Meanwhile, HLA molecules present numerous distinct peptides, with the intended peptide being only a minute fraction of this repertoire. Notably, the infusion of HLA-A*01/MAGE A3-specific TCR-engineered T cells into patients resulted in fatal toxicity due to cross-reactivity with an HLA-A*01-presenting peptide derived from the muscle protein TITIN (24). Throughout the screening and validation phases of this study, numerous candidates were excluded due to nonspecific activation in functional assays using cell lines of various tissue origins (Figure 1 and Supplemental Figure 1). Analyzing the interactions of the candidate antibody with the human proteome will provide more comprehensive information on its potential off-target effects. Nevertheless, the possibility of off-target toxicity cannot be entirely eliminated until thorough safety studies involving nonhuman primates or clinical trials are conducted. This consideration should remain central to the ongoing development of TCR-like therapy research.

When addressing the challenges posed by the exceedingly low expression level of pHLA, 2 prevailing approaches take center stage: (a) the development of a binder with high affinity and (b) the design of an optimal structure with heightened sensitivity to antigens. Among the candidate abTCRs, 61abTCR exhibits a favorable binding affinity of 11.9 nM, though it is not the highest. Our experience indicates that higher binding affinity is frequently associated with compromised specificity (Supplemental Figure 1). This aligns with the findings of Ahmed et al., who reported that affinity-matured E38 bi-specific antibodies displayed increased cytolytic effects at the expense of specificity (2). In general, achieving both high affinity and high specificity in antibodies is challenging. Recently, Zhao et al. proposed that the regulation of catch bonds between TCR and pMHC can finely tune TCR sensitivity independently of binding affinity (25). This discovery introduces a potentially new strategy for engineering highly functional pHLA-specific T cells.

Regarding receptor design, data derived from the T2-loaded LMP2_426_ model validate our approach, demonstrating that abTCR T cells outperform CAR T cells when responding to lower-density antigens (Figure 3). Although these 2 structures exhibit comparable binding affinities (scFv, 1.98 nM; Fab, 8.36 nM), additional factors likely contribute to the observed functional differences. Notably, 61abTCR T cells initiate natural TCR activation signaling upon A*02/LMP2 stimulation by forming a complex with endogenous CD3, which possessed 10 immunoreceptor tyrosine-based activation (ITAM) motifs. CAR T cells generate activation signaling through the CD3ζ domain, with only 3 ITAM motifs. Therefore, TCR elicits more robust proximal signaling than 4-1BBζ–CAR by recruiting greater levels of tyrosine-protein kinase ZAP70 to the ligated TCR/CD3 complex (26). Similarly, the chimeric STAR receptor, which utilizes TCR machinery, has been reported to demonstrate greater sensitivity than the traditional CAR constructs (27). These findings emphasize the advantages of incorporating endogenous TCR signaling to enhance antigen sensitivity. It is worth noting that, although abTCR and STAR were originally designed to target membrane antigens, their potential will be exerted to a greater extent when targeting pHLA. Furthermore, the mTCR exhibited a low assembling efficacy and compromised specificity, bringing out the issues of exogenously expressed (mimic) TCR chains mispairing with endogenous TCRs. AbTCR capitalizes on the transmembrane and intracellular domains of γδTCR, which can reduce the likelihood of mispairing with endogenous TCR, given that more than 95% of endogenous TCRs are α and β type (28).

Costimulatory signals play an indispensable role in engineered T cell immunotherapy. In the context of endogenous T cell activation, costimulatory signals enhance cytotoxicity, proliferation, and memory differentiation (29). The selection of costimulatory signals also influences the fate of engineered T cells. Commonly employed costimulatory signals include CD28, ICOS, CD27, 4-1BB, OX40, and CD40L, with no universally preferred option. Preferences often vary across diseases and studies (30, 31). In this study, we compared the 2 most frequently used signals, CD28 and 4-1BB, and found that the proproliferation effect of CD28 was less pronounced than that of 4-1BB after 5 or 6 rounds of stimulation. This was also evident in terms of procytolysis and prodegranulation effects. CD28 signaling tended to promote faster but less sustained expansion, consistent with its role in CAR T cell therapy. Conversely, 4-1BB signaling facilitated the formation of central memory T cells, thereby favoring long-term T cell persistence (32).

The method of introducing a costimulatory domain is equally critical. Traditional CAR T cells integrate the costimulatory domain within the synthetic receptor, generating costimulatory and activation signals simultaneously in response to antigen stimulation. In contrast, natural TCR T cells employ separate costimulatory and activation signals. This difference results in CAR T cells displaying atypical immune synapses compared with natural T cells (33). In this study, we introduced costimulatory signals triggered by CD7, a marker expressed on NK and T cells (34). For patients with EBV-LPDs, the prognosis is particularly poor when EBV infects T/NK cells (35). To address this, we selected CD7 to ensure the generation of costimulatory signal, thereby enhancing therapeutic efficacy for patients with EBV-associated T and NK cell LPDs. Interestingly, the CD7-BB costimulatory signal promoted the proliferation of abTCR T cells irrespective of whether the target cells expressed CD7. This phenomenon can be attributed to T cell intrinsic expression of CD7, which may provide reciprocal costimulatory signals. Consequently, Co-abTCR T cell therapy has the potential to address unmet medical needs in EBV-associated NK or T cell diseases, as well as other disorders related to EBV. Generally, the selection of costimulatory signals and the strategy for their incorporation need to be thoughtfully tailored and systematically evaluated for each study.

Limitations also exist in this study. No experimental data confirm the density of pHLA on cell lines used throughout the study or the primary samples. The concepts of high- and low-antigen density of target cells were inferred based on the established knowledge of antigen processing and presenting processes. However, our experimental results seem to support the speculation regarding antigen density. During the study, we attempted to engineer the 61rFc antibody to determine the expression of A*02/LMP2_426_ on cell lines. Unfortunately, negative results were obtained, except for T2 cells loaded with 50 μg/mL LMP2 peptides (Supplemental Figure 10). This finding suggested that the expression of A*02/LMP2_426_ on the cell membrane is extremely low, likely falling below the detection limit of flow cytometry. Future studies could utilize mass spectrometry, which can be utilized to quantify the density of specific pHLA molecules on cell membrane in the future. The endogenously presented antigen xenograft model was inconsistent, transitioning from JVM2 cell line (Supplemental Figure 6) to YT-A*02:01 cell line (Figure 6), both of which are CD7 negative. We switched to YT-A*02:01, derived from YT, to allow for simultaneous inclusion of a negative YT control, thereby confirming the specificity of Co-abTCR T cells and abTCR T cells. Moreover, the antitumor effects of the optimized Co-abTCR T cells in mouse models were not as pronounced as those observed with CAR T cell therapy targeting membrane proteins (21). However, the efficacy of our Co-abTCR T cell remains comparable with the published TCR-like T cells and bispecific antibody targeting other antigens (9, 36). There is still a long way for TCR-like T cells to be applicable and effective in clinical settings. This study provides valuable insights into the engineering of TCR-like T cells, particularly in designing optimal receptor structures. TCR-like T cell therapy, combined with strategies of regulating the antigen presenting process, may offer a more effective therapeutic approach for combating cancer.

## Methods

### Sex as a biological variable.

In the whole study, sex was not considered as a biological variable. To minimize the effect of aggressive interactions among cohoused mice on experimental results, we used female animals in the whole study.

### Study design.

This study was designed to engineer A*02/LMP2-specific T cells for the treatment of EBV-associated diseases. Firstly, antibodies targeting A*02/LMP2 were identified using phage display technology. The candidate antibodies underwent rigorous evaluation, including binding assays at the phage level and functional assessments at the T cell level. Throughout these experiments, a benchmark, E38, was employed as a control. Ultimately, clone 61 was chosen for further investigation. Secondly, we developed abTCR T cells based on clone 61 and confirmed their specificity, killing efficacy and degranulation level in vitro in various tumor cell lines and primary samples. Furthermore, we compared the performance of the abTCR T and CAR T when encountering target cells with varying antigen densities of the A*02/LMP2. Third, upon facing challenges in mouse experiments, the abTCR T cells were reengineered by incorporating a costimulatory CAR structure to provide 4-1BB or CD28 signals upon CD7 stimulation. The detailed construct arrangements of CAR, abTCR, and costimulatory abTCR were depicted in Supplemental Figure 5E and Supplemental Figure 7A. In vitro assessments were conducted to evaluate the proliferation, antitumor activity, and degranulation capacities of these modified T cells on target cells expressing CD7 or not. The abTCR-BB T cells were selected as the optimal Co-abTCR format. Finally, the functionality of abTCR T cells and Co-abTCR T cells was evaluated in tumor xenograft mouse models, including an artificially overexpressed antigen model and an endogenously expressed antigen model. In the whole study, sex was not considered as a biological variable.

### Cell lines and culture.

T2, JVM2, SNK6, U266, NALM6, 293T, JEKO1, HCT116, HEPG2, MDA-MB-468, PANC1, NCI-H460, OVCAR3, BV173, YT, RAJI, K562, THP1, JURKAT, OCI-AML3, and CCRF cell lines were purchased from the American Type Culture Collection and verified negative for Mycoplasma. Detailed information regarding tissue origin, the status of EBV infection, A*02 expression, and types of culture medium are described in Supplemental Table 1. Firefly LUC ffLuc cell lines were created by transducing parental cells with lentivirus encoding ffLuc and puromycin-resistant genes and were cultured in the media with puromycin 1 μg/mL (Gibco) (most cell lines) or 0.4 μg/mL for YT-ffLuc (NK/T cell leukemia) and SNK6-ffLuc (NK/T cell lymphoma). A*02/LMP2-overexpressed cell lines (based on A*02 positive Jeko1 or U266) were established using the PresentER Minigene system with a structure of ER signal peptide, LMP2_426_, and terminal codon (Supplemental Figure 4) (37). The overexpressed LMP2_426_ peptides directly enter endoplasmic reticulum and noncovalently bind to A*02, leading to a higher level of A*02/LMP2 on the cell membrane. The YT-A*02:01 cell line was established by transducing lentivirus encoding A*02 into YT cells, and the A*02/LMP2 was expressed through natural endogenously antigen processing and presenting procedures.

### Peptide pulsing.

Peptides were purchased from Sangon Biotech and stored at –80°C. T2 cell lines (38) were loaded with specified concentration peptides (LMP2_426_ and WT1_129_) by incubation at 30°C overnight in RPMI-1640 medium supplemented with 2% FBS (both Gibco).

### Screening of pHLA-specific phage clone.

A*02/LMP2-specific antibodies were screened using a fully human scFv phage library containing 1 × 10^11^ diversity (IASO Biotherapeutics) through cell- and bead-panning processes. T2 cells pulsed with LMP2_426_ as positive selection and pulsed with WT1_129_ as negative selection, followed by beads panning using A*02/LMP2 as the target antigen and A*02/ WT1_129_ as the counterpart. Biotinylated-A*02/LMP2 and A*02/WT1_129_ complexes were synthesized by Kactus Biosystems. Enriched phage clones were then validated by ELISA and flow cytometry. In the ELISA, A*02/LMP2 was used as the intended target, with negative controls of A*02/ WT1_129_ and random protein (TNF-α; ACROBiosystems). In the flow cytometry assay, T2 cells pulsed with LMP2 peptides were used as the intended targets, and WT1_129_ was used as the negative control. E38 chimeric IgG antibody was used as a benchmark in both assays. Phage clones, which bind to the targets but not the negative controls in both ELISA and flow cytometry assay, were selected and sequenced.

### Assembling efficacy determination.

PE-conjugated tetramer of A*02/LMP2_426_ was used to detect the abTCR expression through flow cytometry, and the assembling efficacy was calculated as the percentage of abTCR^+^ within the EGFR^+^ T cell population. Mock T cells and the Jurkat cell line were used as quality controls to verify the specificity of the assay.

### LUC reporter gene assay.

The Jurkat-NFAT-ffLuc cell line was established by transfection of Jurkat cell lines with lentivirus encoding NFAT-response element-controlled ffLuc. The abTCR plasmid was subsequently electrotransfected into Jurkat-NFAT-ffLuc cells. Transfection efficacy was evaluated 48 hours later by flow cytometry. Beyond 72 hours after transfection, Jurkat-NFAT-ffLuc-abTCR cells were cocultured with the targets overnight (ratio of 1:1). Upon antigen binding of abTCR, the influx of calcium activates NFAT, eventually initiating the expression of ffLuc (39). LUC activity, indicative of abTCR signaling, was detected using the ONE-Step Luciferase Assay System (BPS Bioscience).

### Lentivirus production and engineered T cell manufacture.

Genes encoding CAR, abTCR, or Co-abTCR were synthesized and cloned into transfer plasmids individually. Lentivirus production was accomplished in Lenti-X 293T cells using psPAX2 and pMD2.G plasmids and Lipofectamine 3000 Transfection Reagent (Invitrogen). The supernatant containing packaged virus was collected at 48 and 72 hours after transfection, and was concentrated by centrifugation for 1 hour at 4,100*g*, 4°C using the Amicon Pro Purification System with 100 kDa Amicon Ultra-0.5 Device (Merck). The virus was stored at –80°C.

T cells were isolated from merchandised PBMCs using anti-CD3 microbeads (Miltenyi Biotec) and activated by Dynabeads Human T-Activator CD3/CD28 (Thermo Fisher Scientific) on day 0. T cells were transduced with lentivirus on day 1. After 24 hours, the cells were resuspended in a fresh medium. Later, transduced and nontransduced (mock) T cells (1 × 10^6^ to 2 × 10^6^ cells/mL) continuously expanded in CTS OpTmizer medium with 10% FBS (Thermo Fisher Scientific), 200 IU IL-2 (Sigma-Aldrich), and 100 μg/mL l-glutamine (Thermo Fisher Scientific). During 10–18 days of culture, the cells were ready for the functional experiments.

### Binding affinity measurements and epitope identification.

A chimeric antibody with rabbit IgG of the 61 phage clone (61rFc) was produced in CHO-S cells and purified using a protein A column. The binding affinity of 61rFc to A*02/LMP2 was measured by biolayer interferometry using the Octet RED96e system (ForteBio) (40). Alanine-substituted (Ala-substituted) LMP2_426_ peptides at positions 1 and 3–8 were synthesized by Sangon Biotech. Flow cytometry was used to measure the binding ability of 61rFc to T2 cells loaded with 50 μg/mL mutated LMP2_426_ peptides using fluorescence-labeled anti–rabbit IgG antibody. For clone numbers and manufacturer details, refer to Supplemental Table 2.

### CD107A assay.

CAR T cells, abTCR T cells, or mock T cells were stimulated with targets at an E/T ratio of 1:2, overnight, in the presence of 1:100 diluted anti–CD107a-PE/Cyanine antibody and 1:500 diluted monensin. CD107A expression in CD8^+^CAR^+^ or CD8^+^abTCR^+^ T cells was monitored by flow cytometry.

### Cytotoxicity assay.

Cytotoxicity was evaluated through coculturing effector T cells with the targets for 24 hours at the indicated ratios. In total, 2 × 10^4^ tumor cells stably expressing ffLuc were seeded into a 96-well flat-bottom clear plate (Corning, CLS3610). LUC activity was detected after 24 hours of coculture using a Synergy H1 microplate reader (BioTek). Cytolysis was calculated as: Lysis = 1 − luminescence _effector__T__cells__+__targets_/ luminescence _targets_ _only_. A negative value of lysis may be obtained, which implied that the target cell numbers in the study groups increased compared with the control target-only group. For targets without LUC expression, 1 × 10^5^ T2 cells (CFSE-labeled) were added to 96-well U-bottom plates. Cytotoxicity was determined by detecting the proportion of cell death using PI staining (BD Biosciences) and flow cytometry.

### Flow cytometry–based assay.

For samples of cell lines and primary cells, antibodies were added, followed by washing with 2% FBS PBS. After 15 minutes of staining at room temperature and 2 washes, the samples were ready for processing using MACS Quant Analyzer 10 (Miltenyi Biotec). For mouse peripheral blood samples, a 100 μL blood sample was mixed with 900 μL RBC lysis reagent (BD Biosciences) and incubated for 10 minutes. Rat-originated anti–mouse CD16/CD32 (1:500 dilution, BD Biosciences) was added after washing with PBS (supplemented with 2% FBS). Following 10 minutes of blocking, samples were prepared for staining, washing, and acquisition. For clone numbers and manufacturer details, refer to Supplemental Table 2.

### Cytokine release assay.

Supernatants from the coculture of effector cells and target cells (IL-2 free, 10% FBS RPMI-1640 medium) were collected for cytokine measurement. The level of IFN-γ was detected by human IFN-γ kit (Cisbio) according to the manufacturer’s instructions

### Repeat antigen stimulation expansion.

Target cells were treated overnight with 1 μg/mL mitomycin C and coincubated with abTCR T cells for 3 days in CTS medium (supplemented with 200 IU IL-2) at a E/T ratio of 1. AbTCR^+^ T cells were calculated based on the total cell number and abTCR^+^ proportion. Stimulated abTCR T cells were restimulated with fresh mitomycin C-treated targets (ratio of 1:1) and the expansion of abTCR^+^ T cells was calculated. The stimulation was repeated over 5–6 rounds spanning approximately 24 days and typically beginning 6–8 days after T cell isolation. The cumulative expansion of abTCR^+^ T cells was obtained from the product of the expansion in each round.

### Mouse xenograft models.

Female NCG (NOD/*prkdc^–/–^IL2Rg^–/–^*) mice aged 6–8 weeks were injected with tumor cells, 2 × 10^6^ JVM2 or 1 × 10^6^ Jeko1-LMP2-GFP-ffLuc (i.v.), or 5 × 10^6^ YT-A*02:01-ffLuc or 5 × 10^6^ YT-ffLuc (s.c.). After inoculation (3 days for JVM2 and Jeko1-LMP2, 14 days for YT-A*02:01 and YT), mice were divided into different groups. In total, 4 × 10^6^ effector cells (abTCR T cells or Co-abTCR T cells), mock T cells, or PBS were delivered via the tail vein. Peripheral blood samples were collected through the submandibular vein, and the tumor burden was checked using animal bioluminescence imaging or tumor volume measurement. Body mass was recorded every 3 days.

### Statistics.

Data analysis and graph plotting were performed using GraphPad Prism Software (version 8.3.0). Data are presented as mean ± SD (or SEM in animal data). Student’s *t* test was used for comparison between 2 groups, and 1-way ANOVA followed by the Dunnett test was used for variance analysis among multiple groups. Survival analysis was performed using Kaplan-Meier curves, and log-rank tests were used to assess differences among groups. All tests are 2 tailed, and statistical significance was assumed at the level of *P* < 0.05.

### Study approval.

Animal studies were approved by the Animal Ethics Committee of ClinBridge-Biotech, Nanjing, China (WKS-AWF-005.02), and conducted in accordance with the IACUC guidelines. All procedures complied with the Association for Assessment and Accreditation of Laboratory Animal Care International regulations. Studies involving clinical samples adhered to the Declaration of Helsinki and were approved by the Ethics Committee of Tongji Medical College, Huazhong University of Science and Technology (2019S949), Wuhan, China. Informed consent was obtained from all participants.

### Data availability.

Further information and requests for the resources and reagents, for academic purposes, should be directed to and will be fulfilled by the corresponding author. The data values of the figures can be found in Supporting Data Values.

## Author contributions

ZD and XH initiated the project, completing the screening and validation of TCR-like antibodies and the construction and functional assay of abTCR T cells. Following their graduation, JC took over the project, validated the existing data, furthered the research of Co-abTCR T cells, and wrote the initial manuscript. Given their respective contributions, JC, XH, and ZD are listed as first authors in descending order of their contributions. JJ and WM contributed to study design. QW, XJ, JL, and M Xie conducted experiments and provided reagents. QL supervised the experiments and analyzed data. GH, GW, XZ, and M Xiao contributed to study design. YZ, GH, and JW made substantial revisions to the manuscript. JW, LH, and JZ proposed the idea. JW, LH, JZ, and TT proposed the study concept and supervised the research. XH, LH, and JZ secured funding.

## Supplementary Material

Supplemental data

Supporting data values

## Version 1 05/22/2025

Electronic publication
