# Supplementary material for: Targeting intracellular LMP2 with costimulatory signal–armed antibody-like TCR T cells
Source: JCI Insight. 2025 May 22;10(10):e178572. doi: 10.1172/jci.insight.178572 (PMC12128957; doi:10.1172/jci.insight.178572)
Supplement: Supplemental data [file jciinsight-10-178572-s041.pdf]

Supplementary Materials

Supplementary figures and legends:

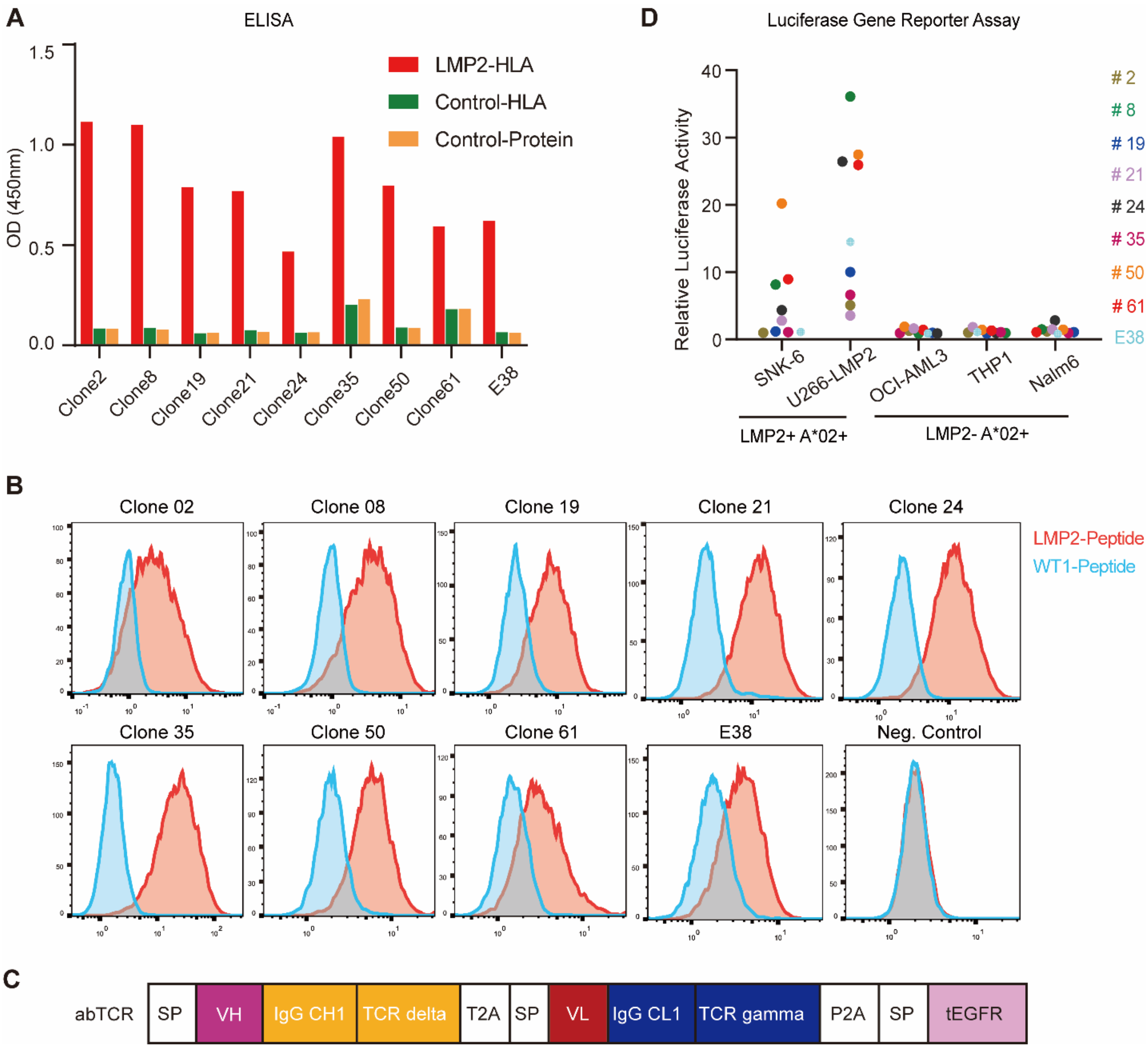

Supplementary Figure 1. Screening of pHLA (A*02/LMP2$_{426}$)-specific binder from phage library. (**A**) Binding of 8 candidate phage clones to HLA-A*02/LMP2$_{426}$ (Red), HLA-A*02/WT1 (Green), and TNF-α (Yellow) by ELISA (n = 1). (**B**) Binding of 8 candidate phage clones to T2 pulsed with LMP2$_{426}$ peptides (Red), WT1 peptides (Blue) by flow cytometry. Previously

validated negative phage clones were used as negative control. (n = 1). (**C**) Schematic construct of the transfer plasmid of abTCR. VH/VL, the variable domain of heavy chain/ light chain. IGHC1/ IGLC1, the constant domain 1 of the heavy chain/ light chain of IgG. TRDC/ TRGC, the transmembrane and intracellular domains of the delta/ gamma chain of TCR. The two chains, *VH-IGHC1-TRDC* and *VL-IGLC1-TRGC*, are connected by T2A (self-cleaving 2A peptide). tEGFR is incorporated following a SP (signaling peptide) and P2A (another self-cleaving 2A peptide). (**D**) Luciferase reporter gene assay of the 8 candidate abTCRs in response to different target cells, SNK6, U266-LMP2, OCI-AML3, THP1 and Nalm6. Relative luciferase activity was obtained by dividing the value for the buffer group, 3 parallel repeats were set and the average value was plotted.

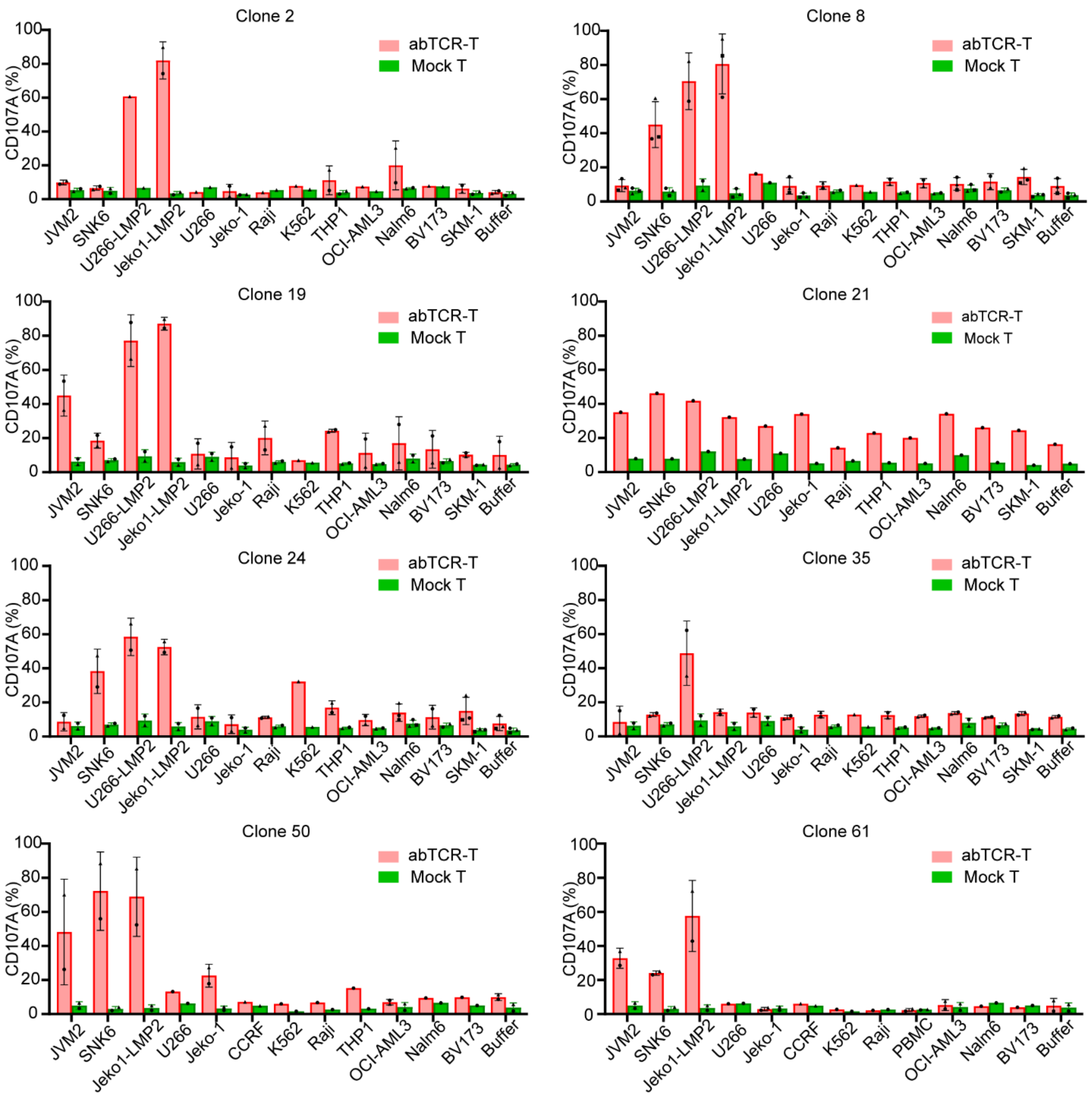

Supplementary Figure 2. Evaluation of candidate clones in abTCR T-cell level by CD107A assay. CD107A expression in $CD8^{+}abTCR^{+}$ T cells cloned from the 8 candidates after overnight stimulation with various target positive cells and target negative cells (n = 1~3).

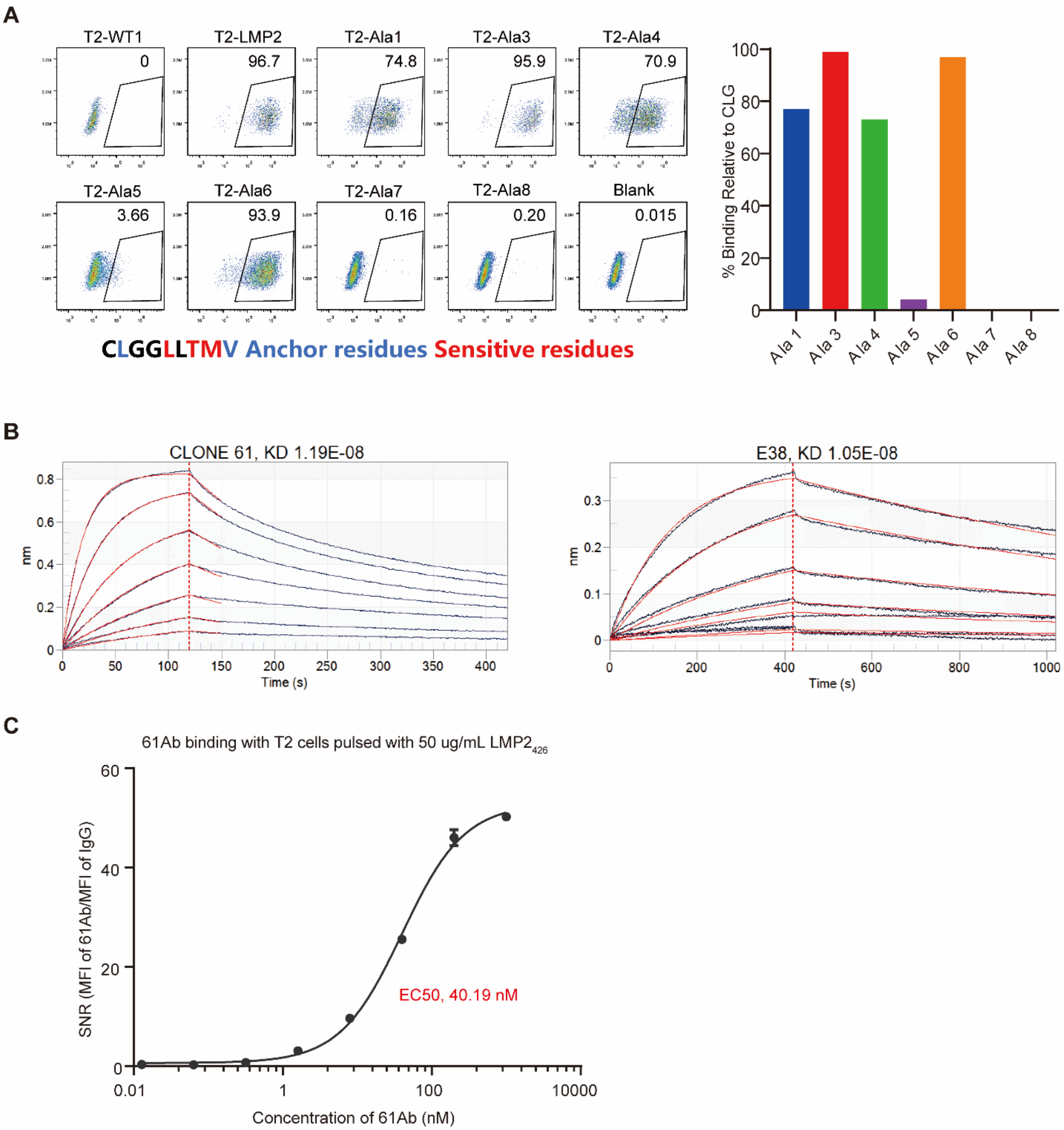

Supplementary Figure 3. Biochemical characteristics of 61rFc. (**A**) Binding of 61 clone chimeric Ab with rabbit IgG (61rFc) to T2 pulsed with alanine-substituted $LMP2_{426}$ peptide. The X-axis of the dot plot represented signal from APC-anti-rabbit IgG. Mutations in anchor residues, specifically at position 2 and 9, hinders the formation of pHLA complex. Sensitive residues to 61rFc are found at position 5, 7 and 8. Position 1 and 4 might also participate in the interaction

with 61rFc (n = 1). (**B**) Binding affinity of 61rFc (11.9 nM) and E38-rFc (10.9 nM) to pHLA (A*02/LMP2$_{426}$) by Biolayer Interferometry. (**C**) The binding of different concentration of 61rFc to T2-pulsed with 50 μg/mL LMP2$_{426}$ by flow cytometry. signal-to-noise ratio (SNR) was calculated from the median fluorescent intensity (MFI) of 61rFc group divided by MFI of IgG group. n = 3. Mean ± SD (standard deviation) and nonlinear fit of curve were plotted.

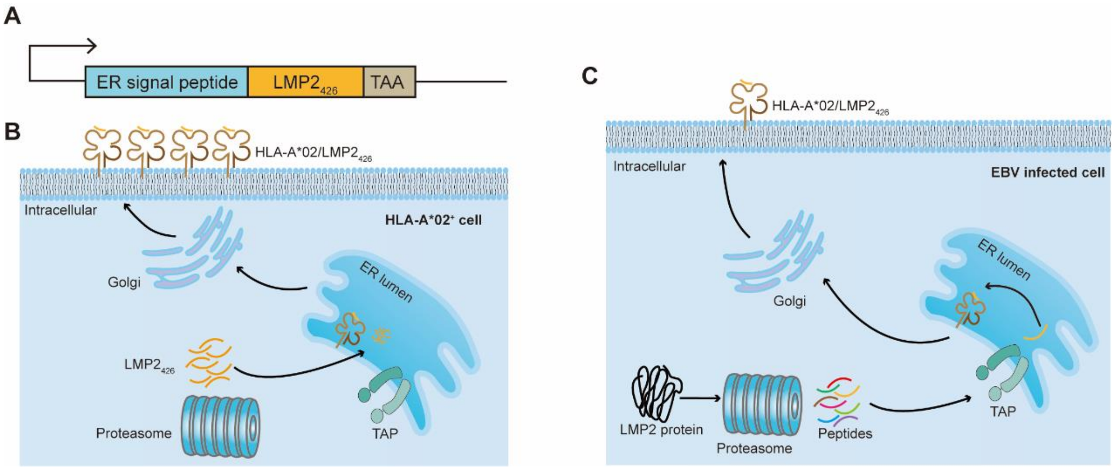

Supplementary Figure 4. Antigen presented artificially by the PresentER method and naturally by the endogenous procedures. (**A**) Schematic construct of the PresentER Minigene. (**B**) Schematic diagram of the working process of the PresentER method. A*02 positive cells are transduced with the lentivirus-encoded the LMP2$_{426}$ PresentER minigenes. The over-expressed LMP2$_{426}$ peptides can directly enter the endoplasmic reticulum (ER) lumen and noncovalently bind to A*02, bypassing degradation by the proteasome, and transportation by the transporter associated with antigen processing (TAP) complex to the ER. The assembled A02/LMP2 complex is then translocated into the cell membrane through Golgi. **C,** Schematic diagram of the endogenous antigen presenting process. In EBV-latency-II/III infected cells, the virus encoding LMP2 protein is first degraded by proteasome, and the produced peptides are then delivered into

the ER lumen through TAP complex. The LMP2$_{426}$ peptides are loaded into HLA-A*02:01 by peptide loading complex to form the A*02/LMP2 complex, and then transported to the cell membrane through the Golgi.

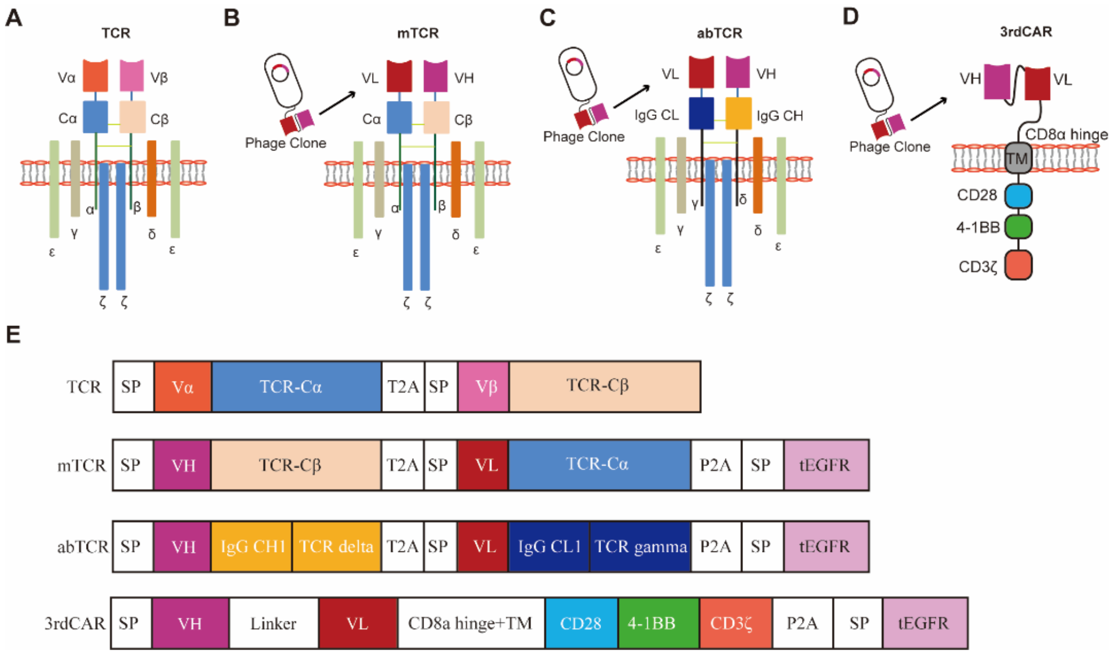

Supplementary Figure 5. The schematic structure of natural αβTCR (A), mimic TCR (B), abTCR (C) and third generation CAR T-cell (D). mTCR and abTCR are comprised of two chains. VH and VL (variable domain of heavy and light chain of antibody) form the binding domain to A*02/LMP2$_{426}$. In the mTCR, the constant regions, transmembrane domains and intracellular domains are the same with αβTCR. In the abTCR, the constant regions are from the constant domain of light chain (IgG CL) and heavy chain (IgG CH) of IgG antibody, respectively. The transmembrane and intracellular domain are the same with the corresponding region of γ and δ TCR. The 3rdCAR is a single chain format. TM, transmembrane domain, CD28 and 4-1BB, costimulatory domains, CD3ζ, activation domain. The corresponding transfer plasmid constructs (E). **mTCR or abTCR**. The two chains of mTCR or abTCR are connected by T2A within the

same transfer plasmid. A tEGFR is also included in the plasmid, connected by P2A following the mTCR or abTCR structure. **3rdCAR.** The following components: VL, linker (2 x G4S repeat), VH, CD8α hinge and CD8α transmembrane domain, CD28 co-stimulatory domain, the 4-1BB co-stimulatory domain, and the CD3 ζ domain are connected directly. A truncated EGFR (tEGFR) is included at the end, connected by P2A sequence.

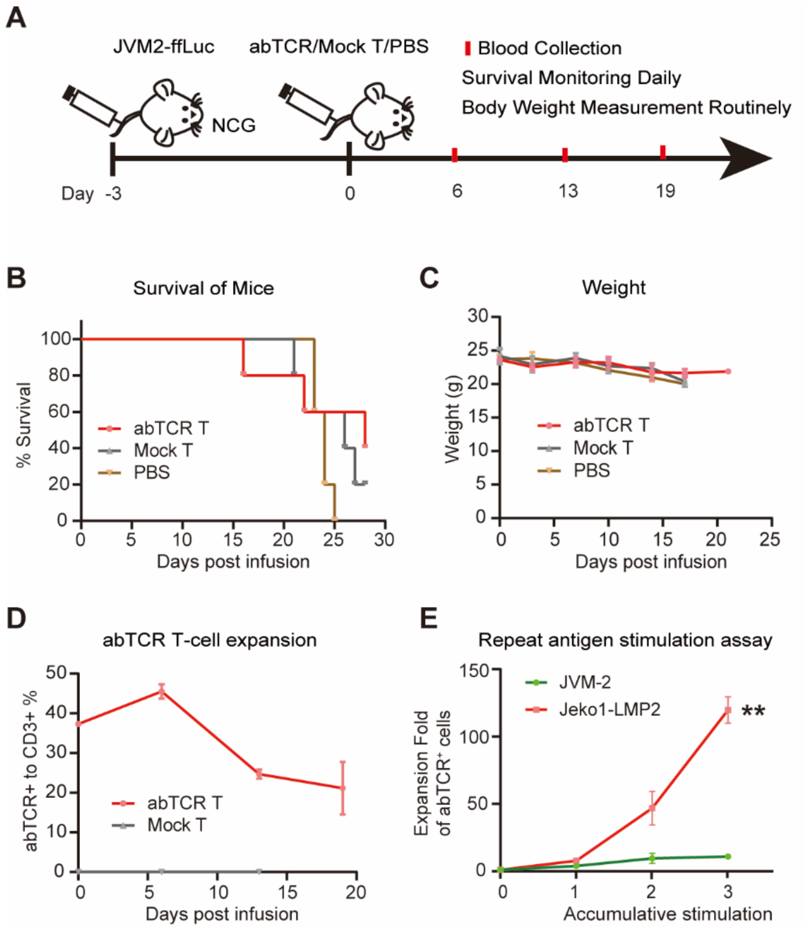

Supplementary Figure 6. The evaluation of abTCR T-cells in JVM2 xenograft NCG mice. **(A)** The animal experimental design: NCG mice intravenously engrafted with 2 x 10^6 JVM2 cells at day -3, and treated with 4 x 10^6 abTCR T-cells, mock T-cells or PBS. The survival and weight of mice was recorded. The expansion of $abTCR^+$ cells was measured weekly. **(B)** The survival of mice in different groups (n = 5). (**C**) The weight of mice in abTCR T-cell treated group (Red line),

Mock T-cell treated group (Grey line) and PBS treated group (Brown line) over time. **(D)** The percentage of abTCR$^+$ to CD3$^+$ cells in peripheral blood over time. **(E)** The accumulative expansion folds of abTCR$^+$ T cells after repeated stimulation of JVM2 cells (Green line) and Jeko1-LMP2 cells (Red line), n = 3. Paired t test was employed. **P < .05*, ***P < .01*, ****P < .001*, *****P < .0001*. Mean and SE were plotted.

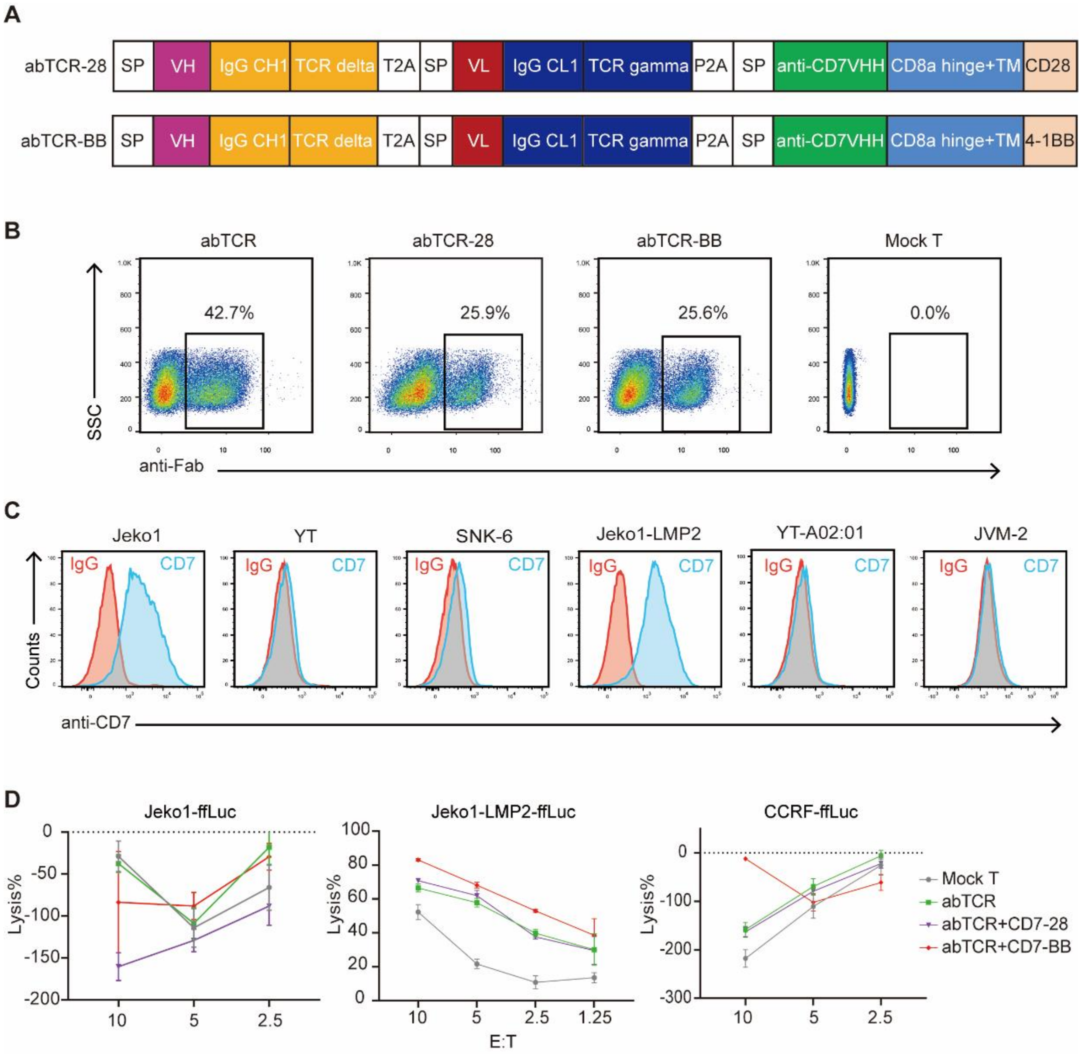

Supplementary Figure 7. The optimization of abTCR. (**A**) Construct arrangements of abTCR-28 and abTCR-BB. Based on the transfer plasmid of abTCR, the tEGFR was replaced by a costimulatory CAR module, consisting of an anti-CD7 VHH, the CD8α hinge and transmembrane

domain, and either a CD28 or 4-1BB costimulatory domain. (B) The expression of abTCR/abTCR-28/abTCR-BB on T-cell determined by flow cytometry from one representative experiment. More than 3 times of experiments were conducted. Allophycocyanin-labeled anti-Fab antibody was used in the detection. **(C)** The expression of CD7 on target cell lines. Anti-CD7 antibody was used in the detection, and isotype anti-IgG antibody was set as control. (**D**) Cytolysis of abTCR, abTCR-28 and abTCR-BB T-cells to Jeko1-ffLuc (pHLA$^-$CD7$^+$), Jeko1-LMP2-ffLuc (pHLA$^+$CD7$^+$) and CCRF-ffLuc (pHLA$^-$CD7+) tumor cells, three technical replicates were set. Mean and SE were plotted.

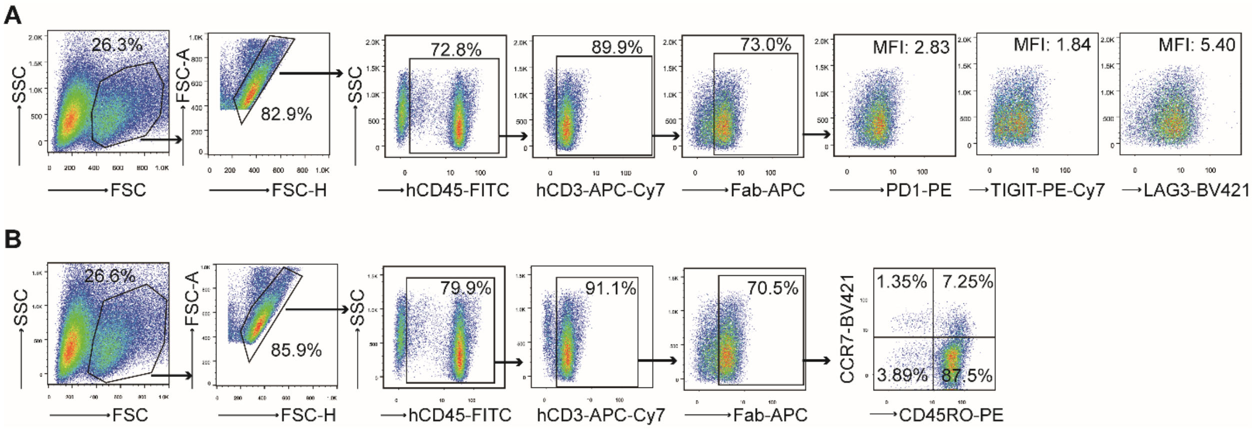

Supplementary Figure 8. Representative flow cytometric gating images of Figure 5, E-G. (A) The gating processes of analyzing the MFI of PD-1, TIGIT, and LAG3 on abTCR$^+$/CAR$^+$ T cells. The percentage of abTCR$^+$/CAR$^+$ to hCD45$^+$ cells was calculated by multiplying 73.0% and 89.9%. **(B)** The gating processes of analyzing the percentage of memory subsets on abTCR$^+$/CAR$^+$ T cells. Naïve: CD45RO$^-$CCR7$^+$, central memory: CD45RO$^+$CCR7$^+$, effector memory: CD45RO$^+$CCR7$^-$, terminally differentiated effector cells: CD45RO$^+$CCR7$^+$.

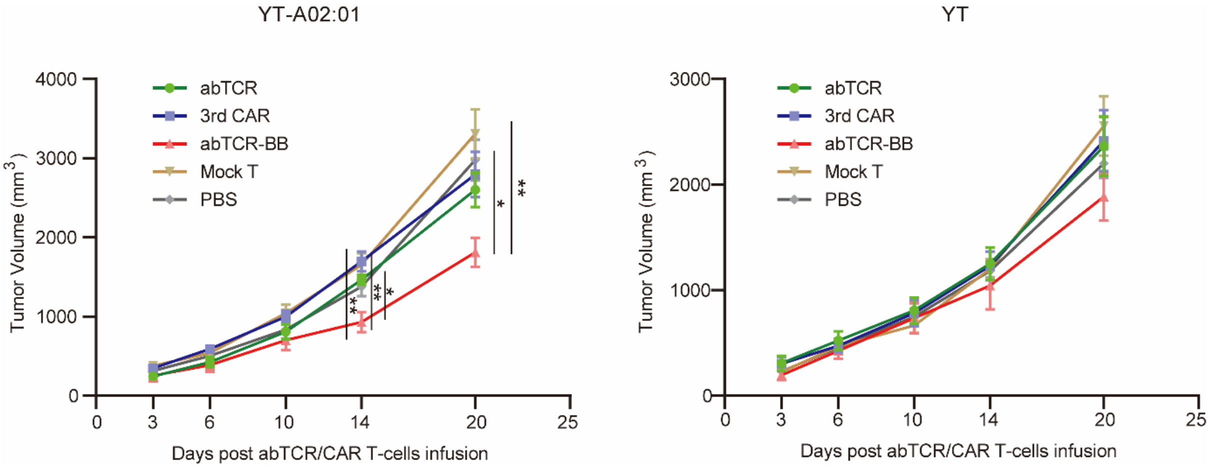

Supplementary Figure 9. The tumor volume of different groups in YT-A*02:01 model (left) and in YT model (right) over time. The mean and SE were plotted. n = 5, and one-way ANOVA with Bonferroni's correction for multiple comparison was used. *$P < .05$; **$P < .01$.

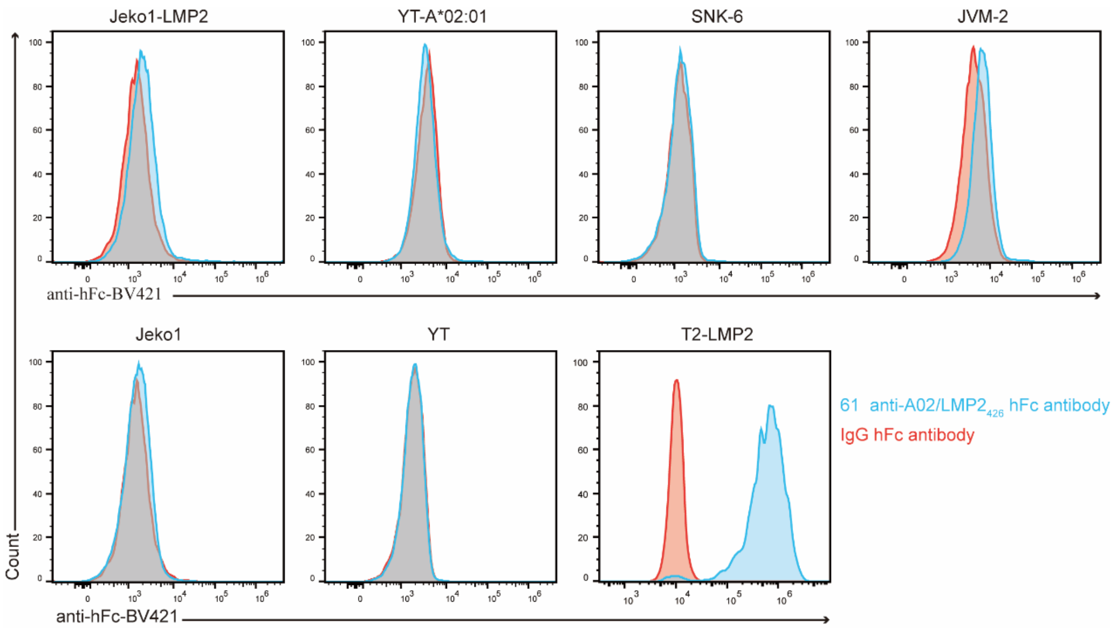

Supplementary Figure 10. The expression of A*02/LMP2 in the cell lines detected by FMC using 61 hFc antibody. Anti-A02/LMP2 hFc antibody (Green line) or IgG hFc antibody (Red line) was used to lable the targets. After wash, anti-hFc-BV421 antibody was used to detect the signal through flow cytometry. T2-LMP2 means T2 cells loaded with 50 µg $LMP2_{426}$ peptides.

Supplementary Tables:

Supplementary table 1: Information of cell lines and primary cells

Name	Origin	HLA-A*02	LMP2	Culture Medium
SNK6	NK/T cell lymphoma	Y	Y	RPMI-1640 + 10% FBS + IL-2
YT	NK/T cell leukemia	N	Y	
YT-A*02:01[#]	NK/T cell leukemia	Y*	Y	
293T	Embryonic kidney	Y	N	DMEM + 10% FBS
HCT116	Colon	Y	N	
HEPG2	Liver	Y	N	
MDA-MB-468	Breast	Y	N	
PANC1	Pancreas	Y	N	
NCI-H460	Lung	Y	N	
OVCAR3	Ovary	Y	N	
T2	T1 transformed	Y	N	RPMI-1640 + 10% FBS
JVM2	EBV transformed B cells	Y	Y	
RAJI	Burkitt lymphoma	N	Y	
U266	Multiple myeloma	Y	N	
U266-LMP2[†]	Multiple myeloma	Y	Y[†]	
Jeko1	Mantle cell lymphoma	Y	N	
Jeko1-LMP2[†]	Mantle cell lymphoma	Y	Y[†]	
NALM6	Acute B cell leukemia	Y	N	
BV173	Chronic myeloid leukemia	Y	N	
K562	Chronic myeloid leukemia	N	N	
THP1	Acute myeloid leukemia	Y	N	
JURKAT	Acute T cell leukemia	N	N	
OCI-AML3	Acute myeloid leukemia	Y	N	
PBMC	Primary PBMC	Y/N	Y/N	RPMI-1640 + 10% FBS

[#]: HLA-A*02:01 was transduced into YT cells by lentivirus transfection;

†: $LMP2_{426}$ peptides was over-expressed by PresentER minigene system, which can be directly presented by HLA-A*02, expected to generate a high density of A*02/LMP2 on HLA-A*02^{+} cell membrane;

FBS: Fetal Bovine Serum; PBMC: Peripheral Blood Mononuclear Cell; Y: Yes; N: No.

Supplementary Table 2. Antibodies used in this study

Antibodies	Fluorescence	Clone	Company
anti-EGFR	APC	AY13	Biolegend
anti-Fab	APC	Polyclonal	Jackson
anti-CD8	BV421/FITC	SK1	Biolegend
CD107A	PE-Cy7	H4A3	Biolegend
anti-PD1	PE/APC-Cy7	A17188A	Biolegend
anti-LAG3	BV421/PE	11C3C65	Biolegend
anti-TIGIT	PE-Cy7	A15153G	Biolegend
CD45RO	PE	UCHL1	Biolegend
CCR7	BV421	150503	Biolegend
anti-hCD45	FITC	HI30	Biolegend
anti-hCD3	APC-Cy7	OKT3	Biolegend
PI	/	/	BD, USA
